# Spatiotemporal dynamics of the host-tumor metabolic interface: Implications for precision nutritional oncology

**DOI:** 10.1016/j.gendis.2026.102345

**Published:** 2026-07-02

**Authors:** Mao Li, Canhua Huang, Shuguang Yu

**Affiliations:** a School of Medical and Life Sciences, Chengdu University of Traditional Chinese Medicine, Chengdu, Sichuan 611137, China; b School of Health and Rehabilitation, Chengdu University of Traditional Chinese Medicine, Chengdu, Sichuan 611137, China; c Department of Biotherapy, Oxidative Stress Research Center, Cancer Center and State Key Laboratory of Biotherapy, West China Hospital and West China School of Basic Medical Sciences and Forensic Medicine, Sichuan University, Chengdu, Sichuan 610041, China; d Frontiers Medical Center, Tianfu Jincheng Laboratory, Chengdu, Sichuan 610041, China; e School of Acupuncture Moxibustion and Tuina, Chengdu University of Traditional Chinese Medicine, Chengdu, Sichuan 611137, China

**Keywords:** Gut microbiome, Interorgan crosstalk, Metabolic plasticity, Nutritional oncology, Precision nutrition, Tumor microenvironment

## Abstract

Beyond somatic initiation, cancer progression is governed by a multidimensional systemic metabolic architecture. This permissive macroenvironment, shaped by systemic nutrient fluxes, endocrine networks, and microbial co-metabolites, sustains tumor bioenergetics and immune evasion. Bridging the current translational gap requires decoding the spatiotemporal reciprocity between diet-induced metabolic shifts, tumor microenvironment plasticity, and genotoxic therapy responses. In this review, we deconstruct the host-tumor metabolic interface through a systems biology framework, tracing the biotransformation of macro-dietary inputs into subcellular oncogenic and immunological signals. We move beyond traditional nutritional epidemiology to define a precision nutritional oncology paradigm that leverages high-resolution multi-omic biomarkers to track real-time systemic flux, the spatial ecosystem of the microbiome as a localized metabolic bioreactor, and context-dependent metabolic regulation to exploit transient tumor vulnerabilities. We emphasize the need for precise spatiotemporal calibration, particularly during acute refeeding windows, to prevent paradoxical therapy resistance or accelerated cachexia. Finally, we envision a future in which precision nutrition is seamlessly integrated into oncologic care, powered by artificial intelligence and digital gut twins for *in silico* modeling. By incorporating the broader chemical exposome and addressing structural socioeconomic determinants through frameworks such as the Planetary Health Diet, we outline a roadmap toward global health equity and enhanced metabolic resilience in cancer survivorship.

## Introduction

While somatic mutations provide genomic blueprints for malignant transformation, tumor progression and immune evasion require a permissive metabolic milieu.^1^ Once narrowly interpreted through the Warburg effect, which emphasizes a localized dependence on aerobic glycolysis, cancer metabolism is now recognized as a systems-level phenomenon. In this framework, metabolic plasticity is a dynamic trait that is continuously reshaped by host physiology, circadian cues, and nutritional status.^2^^,^^3^ Precision nutritional oncology has emerged within this paradigm as a mechanism-driven discipline that targets metabolic addictions, particularly dependencies on glucose and limiting amino acids, to sensitize tumors to genotoxic and targeted therapies.^4^

Because tumors are embedded within a complex multi-organ network, systemic nutrient fluxes, hormonal signaling, and microbial co-metabolites impose selective pressures that dictate therapeutic outcomes.^5^^,^^6^ We conceptualize the spatiotemporal dynamics of this host-tumor metabolic interface within a dual-axis framework. Spatially, this interface operates across a continuum: from systemic nutrient fluxes orchestrated by metabolic hubs such as the liver and gut through the architectural complexity of tissue niches (including stromal crosstalk and immune infiltration) to the specialized bioenergetics of subcellular organelles such as mitochondria and peroxisomes.^7^ Within this hierarchy, visceral adiposity functions as a potent secondary secretory organ, releasing lipokines that facilitate malignant cell migration and blunt T cell-mediated cytotoxicity.^8^^,^^9^ Temporally, the interface spans the macro-scale evolution of malignant progression,^10^^,^^11^ mid-scale adaptive responses to therapeutic cycles, and the micro-scale influence of circadian rhythms, which synchronize metabolic activity with windows of physiological vulnerability.^12^ In aggressive disease, this temporal coordination is frequently disrupted: hypermetabolic tumors often exhibit dampened or decoupled circadian rhythms, suggesting that metabolic anarchy may confer a competitive advantage over the rhythmic constraints of healthy tissue.^13^ This disruption extends beyond the tumor itself, as primary malignancies such as lung cancer can remotely rewire the circadian metabolome of distal organs, driving rhythmic shifts toward hepatic lipid accumulation that metabolically prime the liver as a pre-metastatic niche and facilitate future colonization.^14^

Viewed through the lens of spatiotemporal dynamics, cancer risk reflects the host's evolutionary and lifetime metabolic trajectory rather than a simple consequence of current adiposity. Temporally, evolutionary evidence suggests that species-wide cancer mortality correlates more robustly with long-term dietary patterns, particularly the oncogenic associations of carnivorous diets, than with body mass alone.^10^^,^^11^ While Mediterranean and plant-based dietary patterns help maintain a prophylactic spatial equilibrium by suppressing systemic inflammation, this evolutionary baseline is being rapidly disrupted by the global transition toward ultra-processed foods and chronic caloric over-nutrition.^15^^,^^16^ This nutritional shift fundamentally remodels the spatial architecture of the host-tumor interface. At the primary site of nutrient absorption, ultra-processed foods compromise the intestinal mucosal barrier, facilitating systemic endotoxemia and establishing a pro-inflammatory milieu conducive to neoplastic initiation.^17^, ^18^, ^19^, ^20^ The temporal accumulation of these risks is most evident in early-onset colorectal cancer, where an early-life exposome, defined by red meat-heavy, fiber-poor diets, reshapes the spatial niche of the gut microbiome. This niche shift enriches for genotoxic polyketide synthase (*pks*^+^) *Escherichia coli*, driving the DNA damage required for accelerated tumorigenesis.^21^, ^22^, ^23^, ^24^, ^25^ Finally, this metabolic crisis scales from the local tumor microenvironment to the systemic macroenvironment. The co-occurrence of obesity and type 2 diabetes exacerbates the interface through chronic hyperinsulinemia and dysregulated adipokine secretion. Consequently, the triglyceride-glucose index, a spatio-metabolic marker of insulin resistance, has emerged as a potent, independent temporal predictor of cancer-specific mortality, capturing the cumulative burden of metabolic dysfunction on the host-tumor interface.^26^

**Figure 1:**
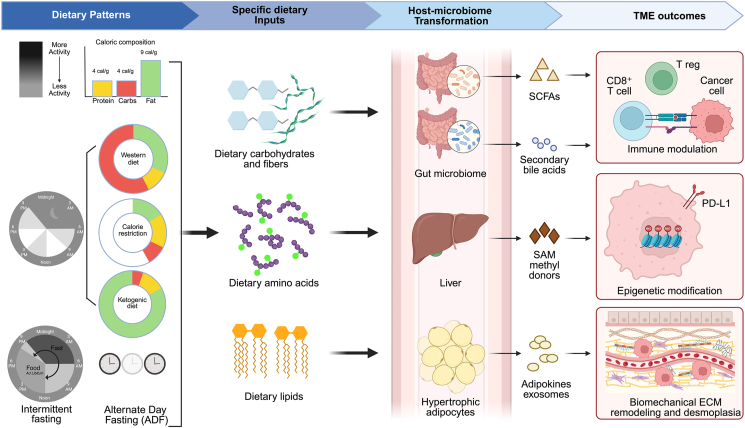
Systems-level integration of dietary inputs and the host-tumor metabolic interface. Macroscopic dietary patterns are defined both by their macronutrient caloric composition (protein, carbohydrates, and fat, with respective energetic values) and their temporal dynamics, as illustrated by 24-h circadian clocks detailing intermittent fasting windows (fasting versus ad libitum feeding synchronized with daily activity levels) and alternate day fasting (ADF) cycles. These distinct dietary regimens (*e.g.*, Western diet, calorie restriction, ketogenic diet) dictate specific systemic nutritional inputs that traverse a transformative physiological filter orchestrated by the gut microbiome, liver, and adipose tissue. Gut microbes metabolize dietary carbohydrates and fibers into short-chain fatty acids (SCFAs) and biotransform primary bile acids into secondary bile acids, driving local and systemic immune modulation (such as CD8^+^ T-cell and Treg regulation). Concurrently, hepatic metabolism of dietary amino acids supplies S-adenosylmethionine (SAM) to fuel epigenetic modifications (*e.g.*, PD-L1 methylation) in malignant cells, while hypertrophic adipocytes process dietary lipids to release distinct adipokines and exosomes that promote biomechanical extracellular matrix (ECM) remodeling and desmoplasia. Abbreviations: cal/g, calories per gram; AM, ante meridiem; PM, post meridiem; ADF, alternate day fasting; Carbs, carbohydrates; SCFAs, short-chain fatty acids; CD8, cluster of differentiation 8; Treg, regulatory T cell; SAM, S-adenosylmethionine; PD-L1, programmed death-ligand 1; Me3, trimethylation; ECM, extracellular matrix.

The clinical urgency of this paradigm shift is underscored by the observation that metabolic syndrome and dietary risks now rival tobacco as primary drivers of cancer-related disability-adjusted life years.^27^^,^^28^ Consequently, mapping the spatiotemporal trajectory of dietary inputs, from initial enzymatic processing to their terminal distribution within the tumor microenvironment (TME), is essential for overcoming therapeutic resistance.^29^^,^^30^ The maturation of nutritional oncology necessitates a transition from observational epidemiology to a rigorous systems-biology framework (Fig. 1). Within this model, macroscopic dietary inputs traverse an individualized physiological bottleneck, an integrated systemic filter shaped by hepatic biotransformation, adipose-derived signaling, and microbial co-metabolism, to generate a personalized metabolome.^31^^,^^32^ At the cellular scale, these metabolites function as signaling molecules that regulate fundamental processes, including epigenetic programming, immune surveillance, and cellular biomechanics.^33^ In clinical settings, these biological insights are translated into targeted nutritional strategies designed to exploit specific vulnerabilities: ketogenic protocols to suppress the insulin-phosphoinositide 3-kinase (PI3K)-mammalian target of rapamycin (mTOR) signaling axis,^34^ periodic fasting to leverage differential stress resistance between malignant and post-mitotic cells,^35^ and selective amino acid restriction (*e.g.*, methionine, serine, or glycine) to disrupt one-carbon metabolism, DNA methylation, and antioxidant defenses.^36^ Critically, these interventions must be synchronized with the host's temporal architecture. Endogenous 24-h biological clocks drive diurnal oscillations in nutrient availability, immune trafficking, and metabolic gene expression, creating rhythmic vulnerabilities that define optimal chronotherapeutic windows for metabolic oncology.^12^ In this review, we dissect the host-tumor metabolic interface, integrating multi-omic biomarkers and temporal treatment cycles to advance a new frontier of precision nutritional therapies.

## The spatial dynamics of the host-tumor metabolic interface: From organ to subcellular levels

The host-tumor metabolic interface can be conceptualized along a continuous spatial gradient, extending from systemic macro-organs to the competitive microenvironment of the tumor. This interface begins with the systemic coordination of nutrient processing, whereby the gastrointestinal tract orchestrates the degradation and absorption of macromolecules, and the liver functions as a central metabolic hub, integrating nutrient fluxes to balance storage (*e.g.*, glycogenesis) and systemic release. Within the TME, malignant cells act as nutrient sinks, outcompeting infiltrating immune cells and stromal elements to limit substrates such as glucose and amino acids.^5^^,^^37^ Whereas healthy cells predominantly rely on mitochondrial oxidative phosphorylation for efficient adenosine triphosphate (ATP) production, cancer cells hijack this spatial logic by diverting metabolic flux toward cytoplasmic aerobic glycolysis (the Warburg effect) and glutaminolysis, thereby prioritizing rapid biosynthetic expansion over energetic efficiency. Although cancer cells display profound spatial and metabolic plasticity to survive within nutrient-deprived niches, this adaptive rewiring generates discrete, targetable metabolic vulnerabilities that underlie the emerging discipline of precision nutritional oncology.^3^^,^^38^^,^^39^

### Carbohydrate flux: Systemic organ gradients to subcellular reprogramming

The host's systemic control of glucose metabolism functions as a macro-environmental scaffold that profoundly shapes the localized glycolytic program of tumors. While the Warburg effect, the preferential fermentation of glucose to lactate, is a canonical hallmark of cancer, it operates within a broader spatial hierarchy. Beyond ATP generation, high glycolytic flux creates a localized carbon reservoir that supplies intermediates for the *de novo* biosynthesis of nucleotides, amino acids and lipids required for biomass expansion. This metabolic shift has far-reaching consequences for the TME. Massive lactate export via monocarboxylate transporters acidifies the interstitial space to a pH of approximately 6.0–6.5. Spatially, this acidic front acts as a biochemical barrier, promoting extracellular matrix (ECM) degradation and facilitating immune exclusion. Within this low-pH niche, the cytolytic activity of T cells and natural killer (NK) cells is severely impaired, allowing the tumor to maintain an immuno-privileged domain. 40, 41, 42

Critically, this metabolic addiction is not cell-autonomous but sustained by the hijacking of systemic glucose homeostasis. Chronic hyperinsulinemia serves as a primary physiological catalyst. Malignant cells frequently undergo an isoform switch, preferentially expressing fetal insulin receptor isoform A (IR-A) over metabolic isoform B (IR-B). This enables tumors to outcompete healthy tissues for systemic signals, as IR-A has superior mitogenic potency and robustly activates the PI3K/protein kinase B (AKT)/mTOR and Ras/mitogen-activated protein kinase (MAPK) cascades, thereby upregulating glucose transporters such as glucose transporter 1 (GLUT1) and rate-limiting glycolytic enzymes.^43^^,^^44^ Such systemic-to-local signaling shapes early oncogenesis, exemplified by insulin-driven acinar-to-ductal metaplasia in pancreatic ductal adenocarcinoma, which can occur independently of net glucose availability.^45^^,^^46^ In parallel, elevated systemic insulin suppresses hepatic production of sex hormone-binding globulin, increasing the bioavailability of free estrogens and androgens and thereby fueling hormone-dependent malignancies in distal niches.^47^ Precision nutritional interventions, such as glucose restriction or ketogenic diets, aim to disrupt this systemic-local axis. By lowering systemic insulin, these strategies activate AMP-activated protein kinase (AMPK)-sirtuin 1 (SIRT1) signaling and induce the expression of circadian clock genes (*e.g.*, PER), thereby restoring mutation-independent p53 checkpoints and metabolic rhythmicity. In doing so, they effectively reprogram the spatial and temporal dynamics of the host-tumor metabolic interface.^48^^,^^49^

**Figure 2:**
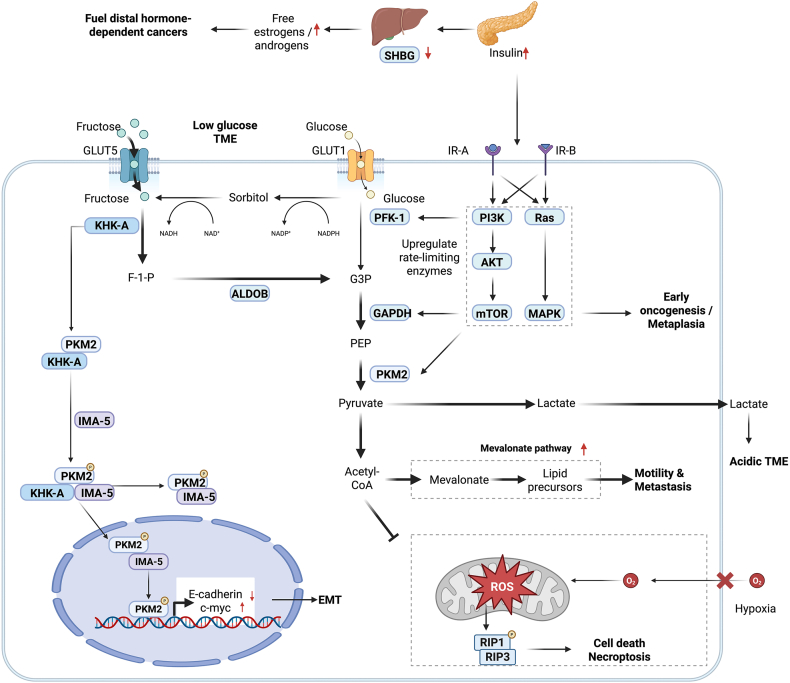
Systemic endocrine hijacking and spatial metabolic rewiring of dietary sugars in the tumor microenvironment. Systemic hyperinsulinemia exerts dual pro-tumorigenic effects across macro- and micro scales. Systemically, excessive insulin suppresses hepatic sex hormone-binding globulin (SHBG) production, elevating circulating free estrogens and androgens to fuel distal hormone-dependent cancers. Locally, insulin engages tumor-expressed insulin receptors (IR-A and IR-B), activating the PI3K/AKT/mTOR and Ras/MAPK signaling cascades. These interconnected networks drive early oncogenesis and upregulate rate-limiting glycolytic enzymes (*e.g.*, PFK-1, GAPDH, PKM2) to sustain anabolic demand. In the glucose-deprived tumor microenvironment (TME), malignant cells undergo a metabolic pivot by scavenging fructose via GLUT5. Fructose catabolism, mediated by ketohexokinase-A (KHK-A) and aldolase B (ALDOB), effectively bypasses the PFK-1 bottleneck to maintain robust glycolytic flux. Concurrently, co-utilization of sugars engages the polyol pathway (via sorbitol), regenerating NAD^+^ to relieve glycolytic constraints. Beyond metabolism, KHK-A directly phosphorylates PKM2, facilitating its IMA-5-dependent nuclear translocation. Nuclear PKM2 acts as a transcriptional co-regulator, repressing E-cadherin and upregulating c-myc to drive epithelial–mesenchymal transition (EMT). Downstream, fructose-derived carbon flux fuels the production of acetyl-CoA, activating the mevalonate pathway to supply lipid precursors essential for cellular motility and metastasis. Furthermore, this robust glycolytic flux acts as a metabolic safeguard, neutralizing hypoxia-induced mitochondrial reactive oxygen species (ROS) and shielding cells from RIP1/RIP3-dependent necroptosis. Last, glycolytic end products undergo conversion to lactate, driving extracellular acidification. Abbreviations: TME, tumor microenvironment; SHBG, sex hormone-binding globulin; IR-A/B, insulin receptor A/B; PI3K, phosphoinositide 3-kinase; AKT, protein kinase B; mTOR, mammalian target of rapamycin; MAPK, mitogen-activated protein kinase; GLUT1/5, glucose transporter 1/5; PFK-1, phosphofructokinase-1; KHK-A, ketohexokinase-A; F-1-P, fructose-1-phosphate; ALDOB, aldolase B; G3P, glyceraldehyde 3-phosphate; GAPDH, glyceraldehyde 3-phosphate dehydrogenase; PEP, phosphoenolpyruvate; PKM2, pyruvate kinase M2; IMA-5, importin alpha-5; EMT, epithelial–mesenchymal transition; ROS, reactive oxygen species; RIP1/RIP3, receptor-interacting protein kinase 1/3; NAD^+^/NADH, nicotinamide adenine dinucleotide (oxidized/reduced); NADP^+^/NADPH, nicotinamide adenine dinucleotide phosphate (oxidized/reduced); Acetyl-CoA, acetyl coenzyme A.

Within the spatial heterogeneity of the TME, glucose availability fluctuates markedly, reaching critical nadirs in poorly perfused necrotic cores or during therapeutic fasting^50^ (Fig. 2). To navigate these glucose-deprived landscapes, malignant cells execute a metabolic pivot, scavenging fructose as an alternative carbon source to sustain anabolic flux. The spatial advantage of fructose lies in its distinct metabolic entry point. Fructose catabolism via ketohexokinase-A (KHK-A) bypasses phosphofructokinase (PFK), the primary rate-limiting step of glycolysis. By circumventing canonical feedback inhibition, fructose-derived flux remains robust even in conditions that would otherwise constrain glycolysis.^51^ This plasticity extends beyond survival, actively rewiring the tumor's metastatic and epigenetic architecture. KHK-A functions as a spatial signal transducer by phosphorylating pyruvate kinase M2 (PKM2) at Ser37, triggering its nuclear translocation. In the nucleus, PKM2 acts as a transcriptional co-activator for pro-migratory gene programs, promoting epithelial–mesenchymal transition (EMT) and conferring anoikis resistance to circulating cancer cells as they exit the primary niche.^52^ In the hypoxic, spatially constrained niches of colorectal cancer, fructose-driven flux also serves as a metabolic safety net, protecting cells from receptor-interacting protein (RIP)-dependent necroptosis and preserving viability under extreme physiological stress.^53^ Moreover, the spatial co-utilization of glucose and fructose activates the polyol pathway, in which sorbitol dehydrogenase (SORD) elevates the intracellular NAD^+^/NADH ratio. This redox shift activates the mevalonate pathway, supplying lipid precursors required for membrane remodeling and cellular motility.^54^^,^^55^ Collectively, the host-tumor fructose interface represents a strategic spatial adaptation that links localized nutrient scarcity to systemic metastatic dissemination.

### Amino acid axes: Spatiotemporal competition and epigenetic remodeling

While normal somatic cells retain biosynthetic plasticity to produce most non-essential amino acids, the host-tumor interface is frequently defined by metabolic addictions or frank auxotropies within the malignant compartment. These vulnerabilities arise from the temporal loss or silencing of key biosynthetic enzymes during tumor evolution, creating a therapeutic window in which dietary restriction can selectively constrain the spatial distribution of metabolites required for nucleotide synthesis, redox homeostasis, and epigenetic programming.^56^, ^57^, ^58^, ^59^

Methionine dependence, or the Hoffman effect, exemplifies a critical spatial tether in amino acid axes. Many tumors cannot proliferate without exogenous methionine, even in the presence of its precursor homocysteine. Restricting methionine flux acutely depletes S-adenosylmethionine pools, the primary methyl donor, precipitating global epigenomic remodeling. This is characterized by DNA and histone hypomethylation and a characteristic S/G_2_ cell cycle arrest.^60^ Beyond intrinsic proliferative control, methionine availability also shapes the tumor immune microenvironment (TIME). Low methionine levels suppress tumor progression by modulating N^6^-methyladenosine (m^6^A) RNA methylation, thereby reducing YTH domain family member 1 (YTHDF1)-mediated translation of immune checkpoints such as programmed death-ligand 1 (PD-L1) and V-domain immunoglobulin suppressor of T cell activation (VISTA).^61^ Tumors reinforce this niche through an oncogenic loop in which methionine drives protein arginine methyltransferase 1 (PRMT1)-catalyzed arginine methylation of Yes-associated protein (YAP), thereby upregulating the solute carrier family 43 member 2 (SLC43A2) transporter to further sequester systemic methionine.^62^ However, precision restriction requires temporal balance, as although it can sensitize tumors to anti-PD-1 therapy, prolonged sulfur deficiency may deplete microbiota-derived hydrogen sulfide, a critical gasotransmitter for CD8^+^ T cell survival.^63^ Restriction of serine and glycine disrupts one-carbon metabolism, a central pathway that couples mitochondrial bioenergetics to nuclear biosynthesis. Serine deprivation in tumor cells induces mitochondrial dysfunction and leakage of mitochondrial DNA (mtDNA) into the cytosol, activating the cyclic GMP-AMP synthase-stimulator of interferon genes (cGAS-STING) pathway, a danger-sensing system that drives type I interferon (IFN) production and recruits effector T cells into the TME.^64^^,^^65^ However, this strategy is a spatial double-edged sword, as tumors can adapt by stabilizing PD-L1 through lactylation, and CD8^+^ T cells themselves require extracellular serine for clonal expansion.^66^^,^^67^ Thus, the temporal deployment of serine restriction must be carefully calibrated to avoid compromising systemic antitumor immunity.

Lineage-specific amino acid dependencies further complicate the host-tumor interface. In *KRAS*-mutant contexts, epigenetic silencing of argininosuccinate synthase 1 (ASS1) creates an absolute dependency on host-derived arginine; depletion of this pool can prime tumor cells for ferroptosis, a regulated form of cell death driven by lipid peroxidation.^68^^,^^69^ Branched-chain amino acids (BCAAs) act as systemic immunometabolic signals. Excess leucine activates leucyl-tRNA synthetase (LARS)^+^ regulatory B cells, which secrete transforming growth factor-β1 (TGF-β1) to locally suppress antitumor immunity.^70^ In asparagine-synthetase (ASNS)-deficient leukemias, l-asparaginase therapy establishes a metabolic asymmetric advantage by spatially starving leukemic blasts while enhancing the metabolic fitness of infiltrating T cells, thereby shifting the competitive balance at the host-tumor interface. 71, 72, 73

### The lipidomic interface: Tissue biomechanics and inter-organ crosstalk

The lipidomic interface functions as a sophisticated regulatory gatekeeper of tumor evolution, in which the structural diversity of dietary fats dictates both tissue biomechanics and the molecular architecture of immune exclusion. Within this framework, lipids are not merely fuel but also serve as spatial scaffolds that reprogramme the TME and temporal signals that coordinate inter-organ metabolic flux.

As systemic glucose and amino acid availability fluctuate, often constrained by the dense, poorly perfused tumor core, malignant cells undergo a temporal shift toward lipid metabolism. This reliance supports membrane biogenesis and generates a protective lipid shield that sustains survival under metabolic stress. Within the spatial hierarchy, saturated fatty acids (SFAs), such as palmitate, act as pro-metastatic cues. Palmitate uptake via carnitine palmitoyltransferase 1A (CPT1α) drives lysine acetyltransferase 2A (KAT2A)-dependent acetylation of NF-κB (p65), installing an epigenetic program that promotes metastatic expansion.^74^ In contrast, stearate can impose a temporal brake on progression by stabilizing mitofusin 1, enhancing mitochondrial fusion, and restoring metabolic rhythmicity in colorectal cancer models.^75^

**Figure 3:**
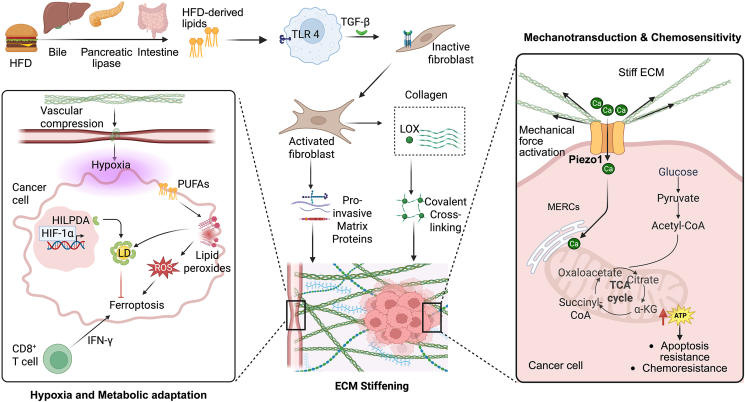
Mechanometabolic and hypoxic adaptations driven by lipidomic and biomechanical signaling in the TME. At the systemic level, a high-fat diet (HFD) undergoes intestinal digestion via bile and pancreatic lipase to release HFD-derived lipids. These overabundant lipids engage signaling cascades (*e.g.*, TLR4) to stimulate TGF-β secretion, which triggers the transition of inactive fibroblasts into an activated state. Activated fibroblasts persistently secrete pro-invasive matrix proteins and collagen. Concurrently, lysyl oxidase (LOX) mediates the covalent cross-linking of collagen, collectively culminating in severe extracellular matrix (ECM) stiffening. Mechanistically (right panel), this enhanced matrix rigidity is translated via the mechanical force activation of Piezo1 mechanosensors into calcium (Ca) influx. Ca traverses mitochondria-endoplasmic reticulum contacts (MERCs) to accelerate the mitochondrial TCA cycle—promoting the metabolic flux from glucose to pyruvate, acetyl-CoA, and subsequent intermediates—thereby boosting ATP synthesis to confer apoptosis resistance and chemoresistance. Concomitantly (left panel), macro-environmental solid stress induces tumor vascular compression, generating severe hypoxia. This hypoxic microenvironment stabilizes HIF-1α, which translocates to the nucleus to upregulate hypoxia-inducible lipid droplet-associated protein (HILPDA). This adaptive metabolic process sequesters polyunsaturated fatty acids (PUFAs) into lipid droplets (LDs), thereby limiting reactive oxygen species (ROS) generation and lethal lipid peroxide accumulation. Consequently, this lipid compartmentalization effectively shields cancer cells from IFN-γ-mediated ferroptosis elicited by CD8^+^ T cells. Abbreviations: HFD, high-fat diet; TLR4, Toll-like receptor 4; TGF-β, transforming growth factor-beta; LOX, lysyl oxidase; ECM, extracellular matrix; Ca, calcium; Piezo1, Piezo type mechanosensitive ion channel component 1; MERCs, mitochondria-endoplasmic reticulum contacts; TCA, tricarboxylic acid; α-KG, alpha-ketoglutarate; Acetyl-CoA, acetyl coenzyme A; ATP, adenosine triphosphate; HIF-1α, hypoxia-inducible factor-1 alpha; HILPDA, hypoxia-inducible lipid droplet-associated protein; LD, lipid droplet; PUFAs, polyunsaturated fatty acids; ROS, reactive oxygen species; IFN-γ, interferon-gamma; CD8, cluster of differentiation 8.

At the macro-tissue level, host lipid status, particularly chronic high-fat diet (HFD) exposure, physically re-engineers the TME. This mechanometabolic transition is characterized by ECM stiffening, altered mechanotransduction, and hypoxic protection. HFDs drive desmoplastic fibrosis and shift the fibroblast secretome toward pro-invasive matrix proteins such as collagen IV and fibronectin, reinforcing ECM stiffness.^76^ The resulting rigid matrix activates Piezo1 mechanosensors, which couple mechanical stress to mitochondria-endoplasmic reticulum contacts (MERCs), thereby linking tissue biomechanics to metabolic signaling (Fig. 3). Vascular compression generates hypoxic microdomains in which HIF-1α upregulates hypoxia-inducible lipid droplet-associated protein (HILPDA), promoting lipid droplet formation, limiting lethal lipid peroxidation, and shielding tumors from immunotherapy-induced ferroptosis.^77^

**Figure 4:**
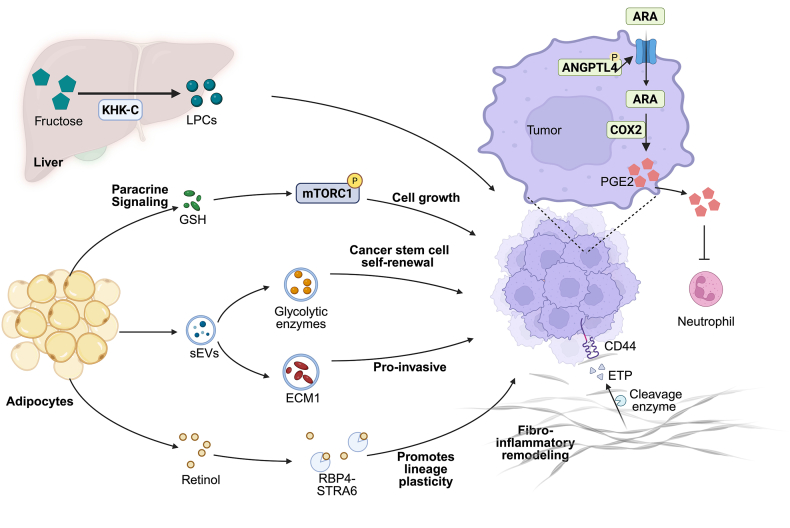
Multi-organ and peritumoral metabolic crosstalk driving tumor plasticity and immune evasion. Dietary fructose intake triggers a systemic-to-local axis by stimulating hepatic ketohexokinase-C (KHK–C) to release lysophosphatidylcholines (LPCs), which are scavenged by distant metastatic clones to fuel membrane biogenesis. Within the tumor microenvironment, hypertrophic adipocytes function as dynamic metabolic donors: adipocyte-derived glutathione (GSH) acts as a paracrine signal that hyperactivates mTORC1 in neighboring cancer cells, enabling sustained cell growth. Simultaneously, the uptake of adipocyte-derived retinol, mediated by the RBP4-STRA6 signaling and transport axis, promotes lineage plasticity and therapeutic resistance in malignant cells. Adipocytes further deploy small extracellular vesicles (sEVs) to shuttle glycolytic enzymes for cancer stem cell self-renewal and ECM1 to foster pro-invasive phenotypes. This metabolic interface also facilitates immune exclusion: obesity-associated signals induce the phosphorylation of ANGPTL4, which enhances the uptake of circulating arachidonic acid (ARA), fuelling a COX2-PGE2 cascade that suppresses effective neutrophil-mediated anti-tumor responses. Finally, fibro-inflammatory remodeling produces endotrophin (ETP), which engages CD44 receptors to further drive malignant behavior and tumor progression. Abbreviations: KHK–C, ketohexokinase-C; LPCs, lysophosphatidylcholines; GSH, glutathione; SCARB2, scavenger receptor class B member 2; ARF1, ADP-ribosylation factor 1; mTORC1, mammalian target of rapamycin complex 1; sEVs, small extracellular vesicles; ECM1, extracellular matrix protein 1; RBP4, retinol-binding protein 4; STRA6, stimulated by retinoic acid 6; ANGPTL4, angiopoietin-like 4; ARA, arachidonic acid; COX2, cyclooxygenase-2; PGE2, prostaglandin E2; ETP, endotrophin; CD44, cluster of differentiation 44.

High dietary fructose intake exemplifies systemic-local coordination at this interface (Fig. 4). Fructose reprograms hepatic metabolism via ketohexokinase-C (KHK–C), stimulating the systemic release of lipid species such as lysophosphatidylcholines (LPCs). These circulating lipids are then scavenged by distant metastatic clones to fuel membrane biogenesis, illustrating how a dietary signal at the gut–liver axis can spatially support colonization in distal niches.^31^^,^^51^ In the peritumoral space, hypertrophic adipocytes function as specialized metabolic donors. In prostate cancer, adipocytes deliver retinol to basal cells via the retinol-binding protein 4 (RBP4)-retinoic acid 6 (STRA6) axis, promoting lineage plasticity and therapeutic resistance.^78^ In obesity-associated breast cancer, adipocytes secrete increased glutathione (GSH), hyperactivating mTORC1 through the scavenger receptor class B member 2 (SCARB2)-ADP-ribosylation factor 1 (ARF1) complex and overriding canonical nutrient-sensing checkpoints to sustain growth in nutrient-poor environments.^79^

Adipose-derived signals further reprogram both systemic and local immunity. Obesity-driven hyperleptinemia phosphorylates angiopoietin-like 4 (ANGPTL4), enhancing uptake of circulating arachidonic acid and fuelling COX-2-dependent PGE_2_ production, which polarizes infiltrating neutrophils toward an immunosuppressive phenotype.^9^^,^^80^ Adipocytes also release small extracellular vesicles (sEVs) that act as spatial delivery vehicles: transferring glycolytic enzymes that support cancer stem cell self-renewal,^81^ shuttling extracellular matrix protein 1 (ECM1) to promote invasion,^82^ and transporting microRNAs that suppress large tumor suppressor kinase 2 (LATS2) and enhance YAP signaling in metastatic niches. The interface is further shaped by stromal accumulation of monoamine oxidases (MAOA/MAOB), which catabolize dietary amines into hydrogen peroxide (H_2_O_2_), amplifying an oxidative, pro-tumorigenic milieu.^83^^,^^84^ A key mediator of this fibro-inflammatory crosstalk is endotrophin (ETP), a collagen VI cleavage fragment that links desmoplastic remodeling to malignant behavior via CD44 engagement.^85^ Even dietary pollutants contribute, inducing exosome-packaged circular RNAs that precondition organotropic pre-metastatic niches.^86^

Importantly, several lipid- and stroma-dependent barriers appear modifiable. Glucagon-like peptide-1 (GLP-1) receptor agonists and structured exercise programs can reprogram pancreatic cancer-associated fibroblasts (CAFs), reduce desmoplasia, and enhance T cell infiltration 87, 88, 89. Neutralization of adipose-derived chemokines such as C-X-C motif chemokine ligand 5 (CXCL5) similarly limits local immune evasion and restores CD8^+^ T cell trafficking into tumors.^90^ Collectively, these findings position the lipidomic interface and its coupled biomechanical and adipose-tumor crosstalk as a promising therapeutic axis for precision nutritional and pharmacologic intervention.

### Microbiome-immune interaction: Inter-organ bioreactors to intracellular signaling

The gut microbiome functions as a highly adaptable, spatiotemporal bioreactor, an effective secondary endocrine organ that mediates the host's metabolic–immune interface (Table 1). This microbial ecosystem dictates the metabolic fate of dietary inputs, biotransforming complex substrates into systemic metabolites that bridge the spatial gap between the intestinal lumen and distal oncogenic niches.^91^^,^^92^

**Table 1:** Pharmacology of host-microbiome cometabolites in oncology.

Dietary precursor	Microbial converters	Bioactive metabolite	Host cellular target/receptor	Immunologic & epigenetic outcomes	Reference
Dietary fiber (inulin, pectin)	Lachnospiraceae, *Bifidobacterium*, *Muribaculum intestinale*	SCFAs (butyrate, acetate)	HDAC inhibition, FOXO1, GPR43/109a, LRP5	Promotes FOXO1-driven stemness in CD127^+^ CD8^+^ T cells; prevents T-cell exhaustion; mitigates muscle wasting in cachexia; decreases cancer stemness via Wnt/β-catenin blockade.	93,209,308
Tryptophan	*Lactobacillus reuteri*, *Enterocloster aldenensis*	Indole derivatives (3-IAA, I3A, IPA)	Aryl hydrocarbon receptor (AhR), Myeloperoxidase (MPO)	I3A activates AhR to promote IFN-γ^+^ CD8 T cells; 3-IAA oxidation downregulates autophagy and degrades ROS-enzymes, boosting chemotherapy/ICI efficacy.	100,101,309,310
Quercetin (flavonols)	Commensal consortia	DOPAC (3,4-dihydroxyphenylacetic acid)	KEAP1/NRF2 axis	Disrupts KEAP1-NRF2 interaction, inducing protective mitophagy in CD8^+^ T cells, maintaining bioenergetic fitness in the TME.	96
High-fat (Animal fats/saturated)	*Clostridium scindens* (7α-dehydroxylating), *Bacteroides*	DCA, LCA (secondary bile acids)	Wnt/β-catenin, G protein-coupled bile acid receptors (FXR)	Recruits immunosuppressive Tregs via CCL28; drives severe colonic epithelial proliferation; 7-oxo-DCA acts as FXR antagonist promoting tumor growth.	97,98,306
High-fat (Unsaturated fats)	*Bifidobacterium breve* CCFM683	CLA (conjugated linoleic acid)	PPAR-γ	Restores tight junctions (ZO-1/Claudin-1) and NF-κB inhibition; induces tumor cell apoptosis in CRC models.	311
Elaidic acid (trans-fatty acids)	*Ligilactobacillus murinus*	Spermidine	p38 MAPK, p53/Bax/Caspase 3	Modulates gut microbiota to produce spermidine; enhances MHC-I antigen presentation and attenuates HCC tumor growth, sensitizing PD-1 blockade.	312,313
Polyamines	*Blautia*, *Odoribacter*	Agmatine	Rnf128	Suppresses Rnf128-mediated β-catenin ubiquitination, hyperactivating Wnt signaling and initiating intestinal dysplasia.	314
Fructose (high sugar diet)	Gut microbiome/Hepatocytes	Acetate/LPCs (lysophosphatidylcholines)	O-GlcNAcylation machinery, Phosphatidylcholine synthesis	Acetate induces O-GlcNAcylation promoting HCC; LPCs are scavenged by CRC cells for membrane synthesis to enhance tumor growth without causing host weight gain.	31,315
Sucralose (artificial sweetener)	Commensal consortia	Induces arginine depletion	T-cell metabolic machinery	Destabilizes gut microbiota leading to reduction in microbiota-accessible arginine; compromises CD8^+^ T-cell function and ablates ICI immunotherapy response.	316
Riboflavin (vitamin B2)	Lachnospiraceae	FAD (Flavin adenine dinucleotide)	FADS2 (fatty acid desaturase 2) in adipocytes	FAD mobilizes mesenteric adipocytes to synthesize PUFAs (like DHA), which increases cytotoxicity of tumor-infiltrating CD8^+^ T cells to enhance anti-PD-1 therapy.	317

Abbreviations: 3-IAA, indole-3-acetic acid; I3A, indole-3-aldehyde; IPA, indole-3-propionate; DOPAC, 3,4-dihydroxyphenylacetic acid; DCA, deoxycholic acid; LCA, lithocholic acid; SCFAs, short-chain fatty acids; TME, tumor microenvironment; HCC, hepatocellular carcinoma; CRC, colorectal cancer; ICI, immune checkpoint inhibitor.

The microbiome-immune axis operates through a finely tuned spatial hierarchy. Microbially derived metabolites act as inter-kingdom signaling molecules that regulate host genetics and epigenetics. Diets rich in plant fibers fuel the fermentation of short-chain fatty acids (SCFAs), such as butyrate and propionate. Spatially, these metabolites traffic to tumor-draining lymph nodes, where they serve as endogenous histone deacetylase (HDAC) inhibitors and drive a forkhead box O1 (FOXO1)-mediated stemness program in tumor-specific CD8^+^ T cells, preserving their long-term anti-tumor fitness.^93^, ^94^, ^95^ Microbial biotransformation of phytochemicals, such as the conversion of quercetin into 3,4-dihydroxyphenylacetic acid (DOPAC), can directly target Kelch-like ECH-associated protein 1 (KEAP1) within the TME, promoting mitophagy and sustaining the bioenergetic competence of infiltrating lymphocytes.^96^

The dietary transition from fiber-rich to Westernized, high-fat patterns mark a critical temporal inflection point in this interface. High-fat diets (HFDs) promote the expansion of 7α-dehydroxylating bacteria (*e.g.*, *Clostridium scindens*), which convert primary bile acids into pro-tumorigenic secondary species such as deoxycholic acid (DCA). Spatially, DCA hyperactivates Wnt/β-catenin signaling and recruits regulatory T cells (Tregs) via the CCL28 axis, facilitating immune evasion.^97^^,^^98^ Specific pathobionts also exert localized structural effects. For example, flagellin from *Desulfovibrio vulgaris* engages leucine-rich repeat-containing protein 19 (LRRC19) on colonic epithelial cells, activating the tumor necrosis factor receptor-associated factor 6 (TRAF6)/transforming growth factor-β-activated kinase 1 (TAK1) cascade and driving EMT.^99^

Microbial processing of dietary amino acids further establishes a cell type-dependent signaling landscape. Commensals such as *Lactobacillus reuteri* metabolize tryptophan into indole derivatives (*e.g.*, indole-3-acetic acid [3-IAA]) that signal through the aryl hydrocarbon receptor (AhR). The spatial consequence is binary: AhR activation in CD8^+^ T cells is required for their reinvigoration, whereas AhR signaling in tumor-associated macrophages (TAMs) promotes immunosuppressive polarization.^100^^,^^101^ Pathogenic taxa can also create metabolic deserts within the TME. *Bacteroides* strains expressing the *bo-ansB* gene deplete local asparagine pools, starving infiltrating T cells and attenuating anti-tumor surveillance.^102^

This interface extends beyond bacteria to include the mycobiome and virome, which operate within distinct anatomical and immunological niches. Fungi such as *Candida* and *Aspergillus* engage in adapter protein caspase-recruitment domain 9 (CARD9) signaling in the aerodigestive tract, promoting IL-1β secretion and the recruitment of myeloid-derived suppressor cells (MDSCs).^103^ The gut virome shapes bacterial ecology through prophage activation; in the context of *H. pylori* infection, this can accelerate early carcinogenesis by altering community structure and function.^104^ Notably, the efficacy and safety of dietary interventions are constrained by baseline microbial architecture. In a dysbiotic, HFD-primed host, highly fermentable soluble fibers can paradoxically induce metabolic overflow, leading to cholestatic liver injury or excessive secondary bile acid production that promotes genomic instability.^105^^,^^106^

Despite this complexity, the microbiome-immune interface is highly modifiable. Administration of *Bifidobacterium* species can deconjugate immunosuppressive taurocholic acid via bile salt hydrolase (BSH) activity, effectively re-spatializing the TME to permit CD8^+^ T cell infiltration.^107^ These interkingdom metabolic flows define a next frontier for microbiota-directed precision nutritional oncology.

### Precision modulation of the redox interface: Spatiotemporal control of homeostasis

Beyond simple nutrient restriction, plant-derived bioactive compounds act as molecular rheostats, selectively modulating metabolic intermediates and functional proteins. At the core of this interface lies the redox paradox, a spatiotemporal bifurcation in which reactive oxygen species (ROS) shift from drivers of early mutagenesis to targetable vulnerabilities in established malignancies.

The host-tumor redox interface exhibits distinct spatial and developmental requirements. In healthy tissues, phytochemicals help maintain redox homeostasis by scavenging ROS, thereby reducing the long-term risk of genomic instability. In contrast, advanced or hypoxic solid tumors often co-opt these same antioxidant defenses to withstand extreme metabolic stress. Precision nutritional strategies aim to invert this protective shield via a controlled pro-oxidant switch. Dietary pro-oxidants such as menadione sodium bisulfite (MSB) selectively oxidize critical cysteine residues, inhibiting class III PI3K vacuolar protein sorting 34 (VPS34) and inducing oxidative cell death.^108^ Pharmacologic doses of vitamin C generate high extracellular concentrations of H_2_O_2_. Once internalized, H_2_O_2_ engages Fenton chemistry with labile metal pools to produce lethal hydroxyl radicals (·OH). This spatially confined killing can be blunted by the selenoenzyme glutathione peroxidase 1 (GPX1). Spatiotemporal restriction of dietary selenium selectively attenuates GPX1 activity in tumors, potentiating vitamin C-induced cytotoxicity while preserving the redox integrity of healthy tissues.^109^

Redox balance is further shaped by inter-organ communication, particularly in hepatic-peripheral flux and the TME. High-dose resveratrol suppresses selenoprotein P (SELENOP), the principal hepatic selenium transporter. Spatially, this retains selenium in the liver, enhancing local antioxidant defenses while creating a systemic deficit in peripheral tumor niches. This selenium desert sensitizes non-hepatic tumors to pro-oxidant regimens such as high-dose vitamin C.^110^ Vitamin E (α-tocopherol) functions as a spatial ligand in dendritic cells (DCs) by binding the phosphatase Src homology 2 domain-containing phosphatase 1 (SHP1), disrupting an intrinsic immune checkpoint. This enhances tumor antigen cross-presentation and coordinates a systemic, T cell-mediated anti-tumor response.^111^

Industrial and thermal processing add a final layer of complexity by generating non-native chemical adducts that reconfigure the redox landscape. Thermal processing yields dietary advanced glycation end products (dAGEs), including N-ε-[carboxymethyl]-l-lysine (CML) and N-δ-[5-hydro5-methyl-4-imidazolon-2-yl]-ornithine (MG-H1), which act as exogenous pro-oxidants and promote a pro-inflammatory milieu conducive to hepatobiliary and gastrointestinal carcinogenesis.^112^ The spatial configuration of the food matrix modulates the absorption and toxicity of these adducts. For example, CML selectively accelerates the progression of hormone receptor-positive breast cancers while sparing benign lesions, highlighting the matrix-dependent sensitivity of specific oncogenic lineages.^113^, ^114^, ^115^

## Temporal dynamics and treatment heterogeneity: Chronological calibration

The maturation of nutritional oncology marks a transition from static diets to dynamic systems biology. The biological impact of a nutrient is no longer viewed in isolation. Nevertheless, it is contingent upon a continuous timeline, from pre-cancerous initiation and active genotoxic stress to metastatic dissemination and survivorship.

### The temporal axis: Synchronizing the fasting–refeeding cycle

The host-tumor metabolic interface is most volatile during the fasting–refeeding cycle, where the precise timing of nutrient reintroduction determines whether an intervention produces therapeutic synergy or paradoxical disease acceleration. Systemic metabolic suppression strategies, such as caloric restriction (CR) and fasting-mimicking diets (FMDs), act as phase-specific modifiers that induce a temporal switch, enhancing stress resistance in healthy tissues while unmasking bioenergetic vulnerabilities in malignant cells.

During the acute fasting phase, the systemic landscape contracts metabolically, creating a window for modality-specific synergies. In triple-negative breast cancer (TNBC), cyclic FMDs synchronize with neoadjuvant chemotherapy to suppress intratumoral glycolysis, resulting in significantly higher pathological complete response (pCR) rates.^116^ This temporal window also enhances the efficacy of cholesterol biosynthesis inhibitors (CBIs),^117^ ferroptosis-inducing agents^118^ and cyclin-dependent kinase 4/6 (CDK4/6) inhibitors^119^ by downregulating mitogenic signals such as leptin and insulin-like growth factor 1 (IGF-1). Carefully controlled 16-h fasting intervals promote the epigenetic reprogramming of CD8^+^ T cells. By modulating isoleucine availability, this phase licenses cytotoxic activity without incurring the systemic risk of cachexia.^120^

**Figure 5:**
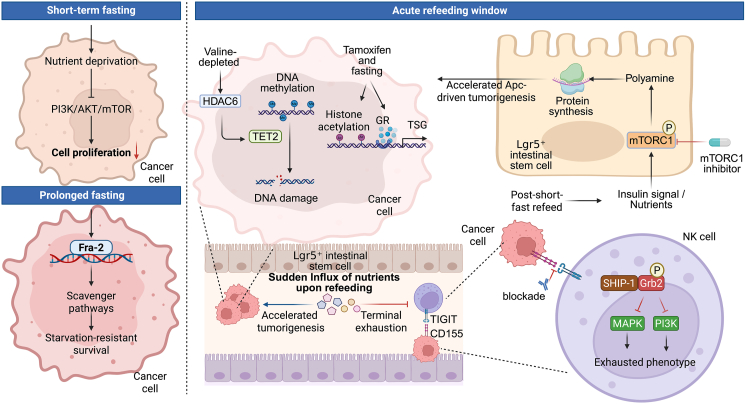
Cellular and molecular dynamics across short-term fasting, prolonged fasting, and acute refeeding windows. The host-tumor interface is profoundly influenced by the duration of nutrient restriction and the subsequent refeeding phase. During short-term fasting (top left), nutrient deprivation inhibits the PI3K/AKT/mTOR signaling pathway, resulting in decreased cancer cell proliferation. In contrast, prolonged fasting (bottom left) can inadvertently activate Fra-2, which triggers scavenger pathways to promote starvation-resistant survival in cancer cells. The transition to the acute refeeding window (right) introduces diverse metabolic and immunological vulnerabilities. Within cancer cells, specific interventions can exploit this phase: valine depletion retains HDAC6 within the nucleus to activate TET2, driving DNA demethylation and inducing targeted DNA damage; concurrently, fasting-induced activation of the glucocorticoid receptor (GR) increases histone acetylation and upregulates tumor suppressor genes (TSGs), thereby potentiating the efficacy of concurrently administered tamoxifen. In the intestinal epithelium (top right), the sudden influx of nutrients and insulin signaling hyperactivates mTORC1 in Lgr5^+^ intestinal stem cells. This fuels polyamine-dependent protein synthesis and accelerates Apc-driven tumorigenesis—a risk that can be intercepted by mTORC1 inhibitors. In the immune microenvironment (bottom right), post-fast refeeding can drive natural killer (NK) cells into terminal exhaustion via the TIGIT-CD155 immune checkpoint. The engagement of this axis recruits SHIP-1 and Grb2 to suppress downstream MAPK and PI3K signaling, enforcing an exhausted phenotype that is reversible via TIGIT blockade. Abbreviations: PI3K, phosphoinositide 3-kinase; AKT, protein kinase B; mTOR, mammalian target of rapamycin; Fra-2, Fos-related antigen 2; HDAC6, histone deacetylase 6; TET2, ten-eleven translocation 2; GR, glucocorticoid receptor; TSG, tumor suppressor gene; Apc, adenomatous polyposis coli; Lgr5^+^, leucine-rich repeat-containing G-protein coupled receptor 5 positive; mTORC1, mammalian target of rapamycin complex 1; NK cell, natural killer cell; TIGIT, T-cell immunoreceptor with Ig and ITIM domains; CD155, cluster of differentiation 155; SHIP-1, SH2 domain-containing inositol 5′-phosphatase 1; Grb2, growth factor receptor-bound protein 2; MAPK, mitogen-activated protein kinase.

The transition from starvation to nutrient surfeit, the acute refeeding window, represents a critical temporal vulnerability (Fig. 5). During fasting, the host translatome is extensively remodeled to prioritize ketogenesis and energy conservation.^121^ A sudden influx of nutrients upon refeeding triggers a sharp, transient burst in mTORC1 signaling. In this window, the regenerative capacity of Lgr5^+^ intestinal stem cells is hyperactivated. Although beneficial for tissue repair, this surge can inadvertently accelerate *Apc*-driven tumorigenesis, converting a nominally protective intervention into an oncogenic driver.^122^ To therapeutically exploit these dynamics, clinical protocols must adopt chronotherapeutic calibration. Without precise timing, prolonged starvation may instead activate transcription factors such as Fos-related antigen 2 (Fra-2) that sustain tumor growth through starvation-resistant programs.^123^

Managing the host-tumor interface thus requires a coordinated, time-dependent strategy. For example, combining TIGIT blockade with glucocorticoid receptor (GR) activation addresses natural killer (NK) cell exhaustion by targeting distinct regulatory nodes. TIGIT blockade lifts an inhibitory checkpoint, restoring immediate effector function.^124^ In contrast, GR activation, despite its traditional association with immunosuppression,^125^ can reprogram NK cells to resist chronic exhaustion by reshaping transcriptional networks.^126^ Navigating the high-risk refeeding window will likely depend on targeted interventions, such as timed mTORC1 inhibitors or valine-depleted formulations. Valine restriction retains HDAC6 within the nucleus, initiating TET2-mediated active DNA demethylation and inducing targeted DNA damage in cancer cells, even as systemic glucose is restored.^127^ Future optimization of these regimens will rely on real-time metabolic monitoring, including genetically encoded biosensors to track polyamine flux and mTOR activity across the refeeding burst.^128^ Conceptualizing nutrition as a phase-specific pharmacologic pulse, rather than a static exposure, offers a strategy to retime the host-tumor interface for maximal clinical benefit.

### Spatiotemporal metastatic escape and the longitudinal immune shift

The cancer experience permanently alters the host's metabolic architecture, establishing a temporal dichotomy in dietary risk. The biological impact of a specific nutritional pattern before diagnosis does not chronologically align with its impact post-diagnosis. Instead, the longitudinal trajectory of diet quality becomes the decisive predictor of survival in aggressive malignancies.^129^^,^^130^ This acquired longitudinal sensitivity is underscored by findings that pro-inflammatory diets significantly elevate cancer-specific mortality only when consumed post-diagnosis, suggesting that the oncogenic event and subsequent treatments fundamentally rewire the host's systemic metabolic response.^131^

As the disease evolves from localized growth to metastatic dissemination, the tumor's metabolic demands undergo a distinct spatial shift. For example, established lung metastases frequently decouple from the metabolic constraints of the primary site, upregulating sorbitol utilization while downregulating glutamine uptake to survive in the pulmonary niche.^132^ Consequently, dietary strategies optimized for the primary tumor may fail in the metastatic setting, necessitating stage-specific nutritional mapping. The long-term impact of therapy further exacerbates this shift. In Stage I-III survivors, the post-diagnostic consumption of ultra-processed foods strictly correlates with elevated long-term mortality, indicating that genotoxic treatments may permanently compromise the host's ability to maintain metabolic homeostasis.^133^^,^^134^

While nutrient deprivation can inhibit primary tumor growth, it also introduces a temporal paradox regarding metastatic vulnerability. Extended starvation windows can induce severe endoplasmic reticulum (ER) stress within malignant cells, triggering the chronological release of tumor necrosis factor-related apoptosis-inducing ligand (TRAIL)-loaded tumor exosomes. Spatially, these exosomes transit to distant organs and exhaust local NK cells, establishing an immunosuppressive pre-metastatic niche that facilitates seeding.^135^ Conversely, this same temporal phase prompts the redistribution of NK cells into the bone marrow. Although this transiently clears the periphery, it facilitates IL-12 priming within the marrow niche, ultimately enhancing the long-term cytolytic functionality of the host's immune defense upon re-entry into the circulation.^136^

The clinical effectiveness of nutritional intervention is strictly governed by acute temporal windows. Surgical trauma induces a rapid, transient state of immunosuppression. Initiating active arginine supplementation specifically during this acute postoperative window is temporally critical for restoring NK cell cytotoxicity and preventing early metastatic recurrence.^137^ Once a patient has chronologically progressed toward cancer cachexia, the timeframe for preventing sarcopenia overrides starvation-based theories. In this state, severe nutrient restriction is detrimental, as the host's metabolic need for anabolic support becomes the primary determinant of survival.^138^ Recognizing these divergent metabolic dependencies across the spatial niches of the disease continuum is paramount for the design of precise, context-dependent nutritional prescriptions that adapt to the evolving host-tumor interface. 139, 140, 141

### Modality-specific synergy: Radiotherapy and chemotherapy

For nutritional interventions to achieve therapeutic synergy, the host-tumor interface must be mechanistically matched to the treatment modality. This metabolic synchronization is essential to avoid biochemical antagonism, where a nutrient might inadvertently protect the tumor or exacerbate host toxicity.

In the context of radiotherapy (RT), the timing of nutrient administration governs the balance between radioprotection of healthy tissue and radiosensitization of the malignancy. Precise timing is a prerequisite for the success of radiotherapy. Fasting or caloric restriction (CR) applied strictly prior to radiation exposure exerts a protective effect on normal tissues, a phenomenon mediated by the transient suppression of hydrogen sulfide generation from sulfur-containing amino acid catabolism.^142^ During active pelvic or head-and-neck RT, oral glutamine serves as a critical substrate for the rapidly dividing cells of the intestinal mucosa, mitigating severe mucositis.^143^ Furthermore, a paradigm shift is emerging regarding dietary fiber. Maintaining a high intake of fermentable fiber during the active timeframe of pelvic RT provides simultaneous mucosal protection and radiosensitization, challenging the historical reliance on low-residue diets.^144^^,^^145^ Conversely, applying nutrient restriction during the radiation cycle can collapse the tumor's glutathione-dependent antioxidant responses, rendering non-essential amino acids (*e.g.*, serine and glycine) conditionally essential under genotoxic stress.^146^

The metabolic shifts induced by systemic chemotherapy require chronological dietary matching to exploit the drug's specific mechanism of action. High-dose antioxidant supplementation is generally restricted to the days of chemotherapy administration to prevent neutralization of the transient, ROS-dependent cytotoxic mechanisms intrinsic to genotoxic drugs.^147^ Precision matching, such as administering phenethyl isothiocyanate prior to cisplatin to deplete glutathione pools or α-tocopherol succinate alongside gemcitabine to induce mitochondrial dysfunction, exploits these timed cellular vulnerabilities.^148^^,^^149^ The toxicity of dAGEs correlates with systemic oxidative stress, specifically at the mid-cycle timeframe of chemotherapy, pinpointing a distinct temporal window where dAGE restriction may be most beneficial.^150^ Coordinating these schedules remains a challenge. For instance, combining time-restricted eating (TRE) with certain chemo cycles can exacerbate genotoxicity over time in specific models, highlighting that fasting is not a universal clinical good but a time-sensitive tool.^151^

Dietary manipulation acts as a chronological regulator of acquired drug resistance, with the duration of exposure dictating the epigenetic outcome. Intermittent FMD can disrupt the localized metabolic protection of metastases (*e.g.*, in the liver), reducing intracellular ATP to impair drug efflux pumps and re-sensitizing the tumor to endocrine or targeted therapies.^152^^,^^153^ Conversely, continuous, long-term exposure to certain bioactives (*e.g.*, phytoestrogens such as genistein) can induce an epigenetic adaptive response. By reducing H3K27me3 methylation and upregulating HER2, chronic exposure can drive acquired resistance to agents such as tamoxifen.^154^ This evidence reinforces the principle that nutritional interventions in oncology must be scheduled as precise, time-sensitive pharmacology rather than static prophylactic measures.

### Lineage and niche specificity: The basis of precision metabolism

The successful deployment of metabolic therapies underscores a fundamental principle: precision nutritional strategies must be tailored to tissue- and cancer-type specificity. Malignant metabolic landscapes are intrinsically shaped by their anatomical niches and developmental lineages. Consequently, a nutritional intervention that starves one tumor may inadvertently provide a compensatory fuel source for another.

For instance, gastrointestinal malignancies, such as early-onset colorectal cancer, are intimately governed by the localized luminal microbiome, making them uniquely responsive to interventions such as fermentable fiber supplementation or heme-iron restriction that directly remodel the mucosal ecosystem to suppress genotoxic microbes.^24^^,^^25^^,^^155^ Conversely, hormone-dependent malignancies, such as estrogen receptor-positive breast or prostate cancers, necessitate systemic strategies that blunt hyperinsulinemia and IGF-1 signaling to prevent visceral adiposity-driven endocrine resistance.^156^^,^^157^ In contrast, highly desmoplastic solid tumors such as pancreatic ductal adenocarcinoma create severe spatial hypoxia and dense stromal barriers; these tumors rely heavily on alternative localized nutrient scavenging (*e.g.*, macropinocytosis of extracellular proteins), uniquely requiring the targeted restriction of specific amino acids such as glutamine or stroma-modulating lipid strategies. 158, 159, 160 Meanwhile, hematological malignancies, which lack a traditional dense solid tumor microenvironment, are exquisitely sensitive to systemic metabolic deprivations in the blood compartment, such as asparagine depletion or transient fasting to block leukemic escape pathways. 161, 162, 163 Recognizing these divergent metabolic dependencies is paramount for transitioning from observational epidemiology to lineage-specific nutritional prescriptions.

**Figure 6:**
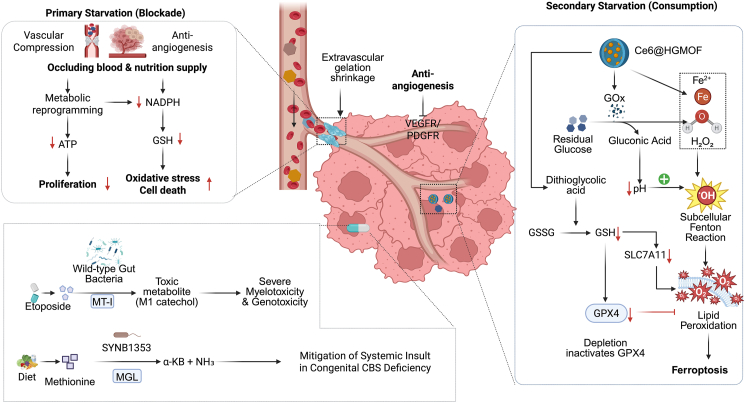
Multimodal spatiotemporal strategies for localized tumor starvation and systemic toxicity mitigation. To physically decouple localized tumor metabolism from host systemic physiology, advanced nanomedicines and synthetic biotics are deployed in a spatially controlled manner. (Primary Starvation): Within the tumor microenvironment (TME), thermo-responsive hydrogels undergo extravascular gelation shrinkage to physically compress intratumoral blood vessels. Concurrently, anti-angiogenic agents block VEGFR/PDGFR signaling pathways. This dual physical and biochemical blockade severely occludes blood and nutrition supply, inducing metabolic reprogramming that depletes ATP and NADPH/GSH, thereby suppressing tumor proliferation and triggering oxidative stress-mediated cell death. (Secondary Starvation): In parallel, a metabolic "consumption" strategy is executed via Ce6@HGMOF nanoparticles. Delivered glucose oxidase (GOx) catalyzes the depletion of residual glucose, generating gluconic acid (which lowers local pH) and H_2_O_2_. The iron-based metal–organic framework (MOF) supplies Fe^2+^ to trigger localized subcellular Fenton reactions, producing highly toxic hydroxyl radicals (·OH). Concomitantly, dithioglycolic acid triggers the depletion of glutathione (GSH), which independently leads to the downregulation of SLC7A11 and the biochemical inactivation of GPX4. These two distinct pathways jointly synergize to accelerate overwhelming lipid peroxidation and target ferroptosis without requiring systemic nutrient deprivation. (Spatial Management of Systemic Toxicity): In the gastrointestinal tract, the gut microbiota serves as a crucial node for toxicity management. While wild-type gut bacteria (via MT-I) detrimentally metabolize oral agents such as etoposide into toxic M1 catechol—leading to severe myelotoxicity and genotoxicity—engineered synthetic biotics (*e.g.*, SYNB1353) act as localized biocatalysts. By expressing specific enzymes (MGL), they actively degrade targeted substrates (*e.g.*, dietary methionine) strictly within the gut lumen into non-toxic byproducts (α-KB and NH_3_). This lumen-restricted degradation accurately replicates the effects of dietary restriction, mitigating systemic insults (such as congenital CBS deficiency) and preventing secondary drug toxicity, ultimately sparing the host's systemic resilience. Abbreviations: ATP, adenosine triphosphate; NADPH, nicotinamide adenine dinucleotide phosphate; GSH, glutathione; GSSG, oxidized glutathione; VEGFR, vascular endothelial growth factor receptor; PDGFR, platelet-derived growth factor receptor; MT-I, methyltransferase-I; MGL, methionine γ-lyase; α-KB, α-ketobutyrate; NH3, ammonia; CBS, cystathionine β-synthase; Ce6, chlorin e6; MOF, metal–organic framework; GOx, glucose oxidase; H_2_O_2_, hydrogen peroxide; ·OH, hydroxyl radical; SLC7A11, solute carrier family 7 member 11; GPX4, glutathione peroxidase 4.

To overcome the systemic toxicity associated with whole-body starvation, advanced nanomedicines aim to physically decouple localized tumor metabolism from host systemic physiology^164^ (Fig. 6). These bio-orthogonal strategies allow for precision control of the host-tumor interface. Thermo-responsive hydrogels can be injected into the TME to embolize localized vasculature, creating a metabolic blackout strictly within the tumor space.^165^ Nanoparticles such as Ce6@HGMOF, which consists of a photosensitizer (Ce6), glucose oxidase, chemotherapy drugs (HCPT) and an iron-based metal–organic framework, simultaneously deliver an H_2_O_2_-generating enzyme (*e.g.*, glucose oxidase) and an iron source from their own framework. This *in situ* co-generation of both key Fenton reaction substrates unleashes a targeted ferroptotic cascade through subcellular Fenton reactions without requiring systemic nutrient deprivation.^166^ Engineering local gut bacteria and synthetic biotics allows for the spatial management of drug toxicity. These localized biosensors replicate the effects of dietary restriction strictly within the gut lumen, sparing the host's systemic resilience.^167^^,^^168^

The future of oncology is shifting from generic recommendations toward chrononutrition and spatial precision. Nutritional interventions are no longer simple lifestyle advice; they function as potent, time-sensitive pharmacologic modifiers. The ultimate objective is to synchronize metabolic inputs with the tumor's rhythmic vulnerabilities. Whether through timed TIGIT blockade during defined fasting phases or the use of mTORC1 inhibitors to blunt the anabolic surge of refeeding, precision oncology must incorporate the multidimensional spatiotemporal dynamics of the host-tumor interface. Only under this integrated framework can we rationally design regimens that synergize with, rather than antagonize, contemporary therapeutic modalities.

## Dietary therapy and precision nutrition: Challenges in clinical translation, epidemiology, and global health

Despite the mechanistic promise revealed by systems biology frameworks, the clinical translation of precision nutritional oncology faces substantial systemic hurdles (Table 2). These arise from the convergence of methodological noise in epidemiologic studies, the structural biology of social disparities, and the intrinsic molecular heterogeneity of the host-tumor interface.

**Table 2:** List of clinical trials on the application of nutritional intervention in cancer treatment.

NCT ID	Phase (N)	Population	Dietary Intervention (Exp *vs.* Ctrl)	Key Outcomes/Status
NCT05040152	N/A (40)	Cancer survivors	Exp: 15-wk Tel-based WL (Diet + PA); Ctrl: Education Brochures	Feasibility: 81.5%; Weight: Exp −4.93 kg *vs.* Ctrl −2.09 kg
NCT04870515	N/A (20)	Prostate Ca.	Exp: RD-led Diet + PA (6mo); Ctrl: 1 RD session	Metabolic: HOMA-IR +4.5% (Exp) *vs.* +42.7% (Ctrl); fat Mass: +1.3 kg (Exp) *vs.* +2.5 kg (Ctrl)
NCT02454517	N/A (117)	Prostate Ca.	Exp: DPP-style WL + Ex; Ctrl: SOC	Metabolic: Fasting Glucose −3.65 (Exp) *vs.* +4.5 (Ctrl); Weight: Exp 90 kg *vs*. Ctrl 99 kg
NCT03987854	Ph 1/2 (29)	PCOS	Exp: VLCKD + PA + Mindfulness	Efficacy: BWL −7.67% (*p* < 0.001); HbA1c −0.21%; Improved QoL/PCOSQ
NCT01482286	Ph 3 (32)	PCOS	Exp: 25% CR ± Metformin ± Ex; Ctrl: No intervention	Terminated early; Confounded data (Ctrl group showed anomalous improvement)
NCT00475982	N/A (44)	Prostate Ca. (Preop)	Exp: Preop WL Program; Ctrl: Control	Weight: WL −3.7 kg *vs.* Ctrl −1.6 kg (*p* = 0.007); Tumor: No sig. diff in Ki67/apoptosis
NCT01697566	Ph 3 (29)	Prediabetic (risk)	Exp: Lifestyle (Diet + Ex) ± Met; Ctrl: Placebo ± Lifestyle	Biomarker: Met + Lifestyle superior for WL (*p* = 0.006); Ki-67 decreased in Met groups
NCT02431676	Ph 2 (121)	Solid tumor survivors	Exp: Coach-led WL *vs.* Metformin; Ctrl: Self-Directed	Biomarker: No sig. diff in IGF-1 or IGFBP3 levels at 6 or 12 mo
NCT00151411	Ph 2 (114)	PCOS	Exp: LCD + Ex ± Metformin; Ctrl: Placebo	Metabolic: Insulin sensitivity Index improved in Met group (+1.9) *vs.* Pbo (−2.7)
NCT02433080	N/A (60)	Uterine Ca.	Exp: “Shape-Up” (Diet + PA); Ctrl: UC	QoL: Net improvement +6.4 pts; Diet quality: Improved in Exp arm
NCT01819233	N/A (38)	Breast Ca. (RT)	Exp: 25% CRD during RT	Adherence: 87.5%; Body Comp: Fat −3.1%, BMI −1.2%; QoL: Stable
NCT05023967	Ph 2 (120)	Breast Ca. (early)	Exp: 16 h IF + Metformin (Pre-op); Ctrl: Usual diet	Status: Active, not recruiting
NCT06399276	N/A (36)	Breast Ca. (Overweight)	Exp: 3 mo IF (5:2) + WL support; Ctrl: N/A	Status: Active, not recruiting
NCT06862323	Ph 2 (75)	Hematological Malig.	Exp: 16:8 TRE (3–6mo); Ctrl: Healthy Ctrl	Status: Not yet recruiting
NCT05764330	N/A (20)	Prostate Ca. (Surv.)	Exp: IF (2 d/wk) *vs.* Cont. Energy Restr; Ctrl: N/A	Status: Results not reported
NCT06804044	N/A (33)	PCOS	Exp: IF (16:8) *vs.* Standard diet; Ctrl: Usual diet	Status: Results not reported
NCT06807775	N/A (45)	PCOS	Exp: TRF (16:8) *vs.* Energy restriction	Status: Not yet recruiting
NCT07322120	N/A (46)	PCOS	Exp: Low fat (CR + IF) + Magnesium; Ctrl: PBO	Status: Not yet recruiting
NCT06824974	N/A (50)	Liver Ca.	Exp: 8–10 h TRE + Plant-Based Diet; Ctrl: Observational	Status: In recruiting status
NCT06315296	N/A (60)	Brain Metastases	Exp: 30 d time-restricted diet; Ctrl: Attention Ctrl	Status: In recruiting status
NCT04288336	Early Ph1	Prostate Ca.	Exp: 16:8 IF post-prostatectomy	Status: Withdrawn (No accrual)
NCT03523377	N/A (28)	Child Ca. Survivors	Exp: Prolonged overnight fasting	Status: Active, not recruiting
NCT04959474	N/A (80)	Breast Ca. (SABR)	Exp: 25% CR (6–12 wk); Ctrl: Standard Recs	Status: In recruiting status
NCT02983279	N/A (49)	Breast/Endometrial Ca.	Exp: Pre-op 25% CR (3–12 wk)	Status: Results not reported
NCT02135562	N/A (11)	Endometrial Ca.	Exp: Protein-sparing Modified fast (WL)	Status: Results not reported
NCT02622711	N/A (262)	Breast Ca.	Exp: WL Diet *vs.* PA *vs.* Combined; Ctrl: Low Intensity	Status: Results not reported
NCT05082519	Ph 2 (240)	ALL (Leukemia)	Exp: Calorie Deficit (Low GL) + Ex; Ctrl: SOC	Status: In recruiting status
NCT06785324	N/A (20)	B-ALL (Induction)	Exp: 10% Calorie Deficit (high Pro)	Status: In recruiting status
NCT03454282	N/A (100)	Breast/Melanoma	Exp: FMD (5-day, q4w)	Status: Recruiting
NCT06610565	N/A (60)	Breast Ca. (AI Tx)	Exp: 5-day FMD/mo; Ctrl: Counseling	Status: Active, not recruiting
NCT06353698	N/A (40)	Leukemia (Chemo)	Exp: FMD + Chemo; Ctrl: Chemo alone	Status: In recruiting status
NCT06438588	N/A (10)	Ca. on Immunotherapy	Exp: 4-day FMD + Nutrition Counseling	Status: In recruiting status
NCT03162289	N/A (150)	Breast/Ovarian Ca.	Exp: Peri-Chemo fasting *vs.* Vegan Diet	Status: Active, not recruiting
NCT01175837	N/A (12)	Pre-Chemo Patients	Exp: Short-term fasting (24–48 h)	Status: Results not reported
NCT05990426	N/A (30)	Ovarian/Endometrial	Exp: Alternate Day fasting (ADF) per-chemo	Status: In recruiting status
NCT00444054	N/A (10)	Metastatic Ca.	Exp: VLC Diet (Keto) for 28 d	Status: Results not reported
NCT03950635	Ph 1 (13)	Melanoma	Exp: Whole food fiber-rich *vs.* Keto	Status: Terminated (Admin complete)
NCT01092247	N/A (40)	Glial Tumors (Recur)	Exp: Individualized Keto Diet; Ctrl: Standard Diet	Status: Not yet recruiting
NCT05938322	N/A (194)	Pelvic Ca. (RT)	Exp: Keto Diet; Ctrl: MedDiet	Status: In recruiting status
NCT04035096	Ph 1/2 (40)	Colon Ca. (Terminal)	Exp: VLCD + IV Vitamin C; Ctrl: SOC	Status: Not yet recruiting
NCT02286167	N/A (25)	Glioblastoma	Exp: Modified Atkins Diet (Intermittent)	Status: Results not reported
NCT06954584	Ph 3 (424)	HRD-neg Tumors	Exp: Low Carb Diet + Fluzoparib	Status: In recruiting status
NCT01802346	N/A (130)	Breast/Prostate Ca.	Exp: Low Carb Diet (3d prechemo); Ctrl: Normal Diet	Status: Active, not recruiting
NCT02149459	Ph 1 (18)	Brain Tumors (Recur)	Exp: Low Carb Diet ± Metformin + Re-RT	Status: Recruiting
NCT03679260	Ph 2 (22)	Prostate Ca. (AS)	Exp: Carb restricted Diet (20 g/d); Ctrl: Unrestricted	Status: Results not reported
Whole dietary patterns, behavioral modification, and digital health				
NCT ID	Phase (N)	Population	Dietary Intervention (Exp *vs.* Ctrl)	Key Outcomes/Status
NCT04534738	Ph 1/2 (33)	Chemo Patients	Exp: MedDiet (Meals provided); Ctrl: UC	Feasible (100% completion); No sig. diff in fatigue; MedDiet score improved
NCT01238172	Ph 3 (478)	Prostate Ca. (MEAL)	Exp: Veg-rich Diet Ed (MEAL); Ctrl: Info Booklet	Veg intake +2.0 serv/d; Time to Progression HR 0.96 (NS)
NCT04920084	N/A (23)	MGUS/SMM	Exp: WFPBD (Meals + Coaching)	High adherence (93%); BMI reduced (34.3–32.0)
NCT02815982	N/A (106)	Ped. Ca. survivors	Exp: NOURISH-T (Family/Beh); Ctrl: Enhanced UC	Improved parent/child weight metrics; reduced SSB/Fast food intake
NCT04396665	N/A (224)	Breast Ca. risk	Exp: Web-App (Diet + PA); Ctrl: No Interv	Exp had higher risk Knowledge scores but higher dropout (26%)
NCT02699983	N/A (35)	Breast Ca. (AA)	Exp: SparkPeople App + Coaching; Ctrl: Waitlist	Self-efficacy increased (+5.59); Weight loss similar (−1.7 *vs.* −2.5 kg)
NCT05887401	N/A (72)	Young survivors	Exp: Traffic Light App + PA; Ctrl: Components	MVPA +61.5 min/wk; Diet quality (HEI) +2.5 pts; reduced Fatigue/Anxiety
NCT04373434	N/A (512)	General (high BMI)	Exp: Healthy Homes (Environment); Ctrl: Mailings	Exp superior in HEI (+3.38), Weight Loss (−6.5 lbs), and home food environment
NCT00416572	N/A (252)	Breast Ca. Survivors	Exp: Nutr. Education *vs.* Psych Ed; Ctrl: UC	Nutr arm sig. improved depression (CES-D) & Physical health *vs.* Ctrl
NCT06016725	N/A (75)	Older survivors	Exp: Nutr Counseling + RT + Ed; Ctrl: Ed only	No sig. diff in Physical function or grip strength; high retention
NCT05746195	N/A (46)	CRC survivors	Exp: SMS Reinforcement for Whole grains	Whole grain intake +22.1%; high system usability (SUS 72.5)
NCT00217490	N/A (621)	Ca. Prevention	Exp: Computer *vs.* Nutritionist *vs.* Combo	All active arms increased F/V intake & reduced fat% *vs.* PA control
NCT03834974	N/A (66)	Ca. (General)	Exp: Social Media Challenge; Ctrl: Sun safety	High retention; sun safety improved in Ctrl; Diet engagement high in Exp
NCT04192071	N/A (139)	CRC Prevention	Exp: Virtual human feedback; Ctrl: Attn Ctrl	No sig. diff in risk Perception; high Info seeking (∼55%)
NCT04947150	N/A (62)	Low Diet adherence	Exp: Location-Triggered Msg + Coach	Sig. reduced Processed Meat/Sodium; Increased goal salience
NCT03719677	N/A (7)	Breast Ca. Survivors	Exp: Habit Development (Diet + PA)	Habit strength (SRBAI) increased 1.6 to 4.8; small pilot
NCT01824498	N/A (18)	Breast Ca. Risk	Exp: LFD *vs.* HFD *vs.* LF-n3	Plasma Estradiol lowest in LFD (35.6) & LF-n3 (39.8) *vs.* HFD (52.8)
NCT04174391	N/A (766)	Breast Ca.	Exp: MedDiet + free EVOO *vs.* LFD	Status: Active, not recruiting
NCT06582615	N/A (60)	TNBC (Breast Ca.)	Exp: MIND Diet (Neuroprotective); Ctrl: Health Ed	Status: In recruiting status
NCT04744974	Ph 2 (28)	MPN (Blood Ca.)	Exp: MedDiet *vs.* DASH Diet	Status: Results not reported
NCT05265819	N/A (80)	Thyroid Ca.	Exp: MedDiet ± Exergaming; Ctrl: Hormone Tx	Status: Not yet recruiting
NCT06236360	N/A (30)	Melanoma (Immuno)	Exp: Tele-MedDiet; Ctrl: Usual diet	Status: Recruiting
NCT04985565	N/A (12)	Prostate Ca. (Preop)	Exp: MedDiet (4 wk preop)	Status: Active, not recruiting
NCT04079270	N/A (200)	Breast Ca.	Exp: Algorithm-based Diet *vs.* Med-LFD	Status: Recruiting
NCT06918054	Ph 4 (42)	ALL (Children)	Exp: MedDiet + Omega-3 (Liver support)	Status: In recruiting status
NCT00003097	Ph 2 (175)	Skin Ca.	Exp: Low fat diet	Status: Active, not recruiting
NCT01913015	Ph 1 (3)	Prostate Ca. (CRPC)	Exp: Low *vs.* High fat Bkfst + Abiraterone	Status: Terminated (PK analysis issues)
NCT04645680	Ph 2 (50)	Melanoma (Immuno)	Exp: Whole food high fiber *vs.* Standard	Status: Active, not recruiting
NCT03489213	N/A (82)	Ca. survivors	Exp: Plant-Based ± Lean Beef (garden-based)	Status: Results not reported
NCT04538482	N/A (112)	Race/Ethnicity study	Exp: DASH *vs.* Standard American Diet	Status: In recruiting status
NCT02129218	N/A (18)	Colon Ca.	Exp: Low GL *vs.* Med GL (Intensity levels)	Status: Results not reported
NCT06635005	N/A (30)	Breast Ca. risk	Exp: Low-EDIH (Inflammatory Index)	Status: In recruiting status
NCT05830487	N/A (24)	PCOS	Exp: Low AGEs (Adv. Glycation Endprod)	Status: Enrolling
NCT03930368	N/A (20)	Insulinoma	Exp: Low GI + Raw Corn starch	Status: Listed as “Insulinoma” (study of diet mgmt)
NCT03066856	N/A (502)	BRCA carriers	Exp: Low Calorie/Protein, Plant-based	Status: Results not reported
NCT04014283	N/A (7000)	Breast Ca. risk	Exp: Selenium-targeted Diet Mod	Status: Active, not recruiting
NCT02788955	N/A (50)	Sarcopenia	Exp: High Protein *vs.* Normal Protein	Status: Results not reported
NCT05356182	N/A (30)	Immunotherapy	Exp: Low Protein *vs.* Standard Control	Status: Recruiting
NCT01687231	N/A (47)	Stem Cell Transpl.	Exp: Nonneutropenic *vs.* Neutropenic Diet	Status: Results not reported
NCT04940468	N/A (5)	C. Difficile Prev.	Exp: High Fiber/Low fat	Status: Terminated (Low accrual)
NCT06212817	N/A (54)	CRC (Preop)	Exp: Personalized fiber diet/Supp *vs.* Ctrl	Status: Recruiting
NCT02954289	N/A (44)	Prostate Ca.	Exp: Cooking Classes & Education	Status: Results not reported
NCT06926972	N/A (11)	Cancer (General)	Exp: NOURISH (F/V + Cooking skills)	Status: Ongoing
NCT01414062	N/A (82)	Breast Ca. Surv.	Exp: “Cocinar Para su salud” (Cooking)	Diet: F/V intake +2.0 serv/d (M6) *vs.* Ctrl
NCT04597151	N/A (13)	CRC survivors	Exp: Group Nutrition Ed (3 sessions)	Status: Results not reported
NCT00082732	Ph 1 (56)	Prostate Ca.	Exp: Low Fat/High fiber + soy + Behavior	Status: Results not reported
NCT00301678	N/A (2520)	Healthy (Unhealthy Diet)	Exp: Behavioral/Prev. Diet + Education	Status: Active, not recruiting
NCT00301691	N/A (2000)	Low-income healthy	Exp: Multi-component Beh. Diet	Status: Results not reported
NCT02279303	N/A (153)	Breast Ca. Surv.	Exp: Anti-Inflammatory Diet (Workshops)	Status: Results not reported
NCT03953157	N/A (9)	Breast Ca. (AI Tx)	Exp: Anti-Inflammatory Diet *vs.* Ex	Status: Results not reported
NCT02965521	N/A (50)	CRC survivors	Exp: Web/SMS Diet Intervention	Status: Results not reported
NCT00120016	N/A (80)	Healthy Women	Exp: MedDiet via Tel-counseling	Status: Results not reported
NCT01570010	N/A (503)	CRC survivors	Exp: 12-mo Intensive Diet (Norwegian)	Status: Active, not recruiting
NCT03410641	Obs (1216)	Ca. Risk (Long-term)	Exp: 5-yr CV risk Diet Counseling	Status: Results not reported
NCT07176455	Obs (438)	Gastric Ca. Surv.	Exp: Behavioral anti-frailty Diet	Status: Enrolling by invitation
NCT05481229	Obs (100)	Child Ca. Survivors	Exp: Targeted Nutr/PA Counseling	Status: Results not reported
NCT04215029	N/A (6)	Prostate Ca. (AA)	Exp: Dyadic (Partner) Nutr + Ex	Status: Active, not recruiting
NCT06379191	N/A (78)	Ovarian Ca. (Chemo)	Exp: WeChat App Nutrition Mgmt	Status: Results not reported
Targeted nutritional supplementation and management of treatment side effects				
NCT ID	Phase (N)	Population	Dietary Intervention (Exp *vs.* Ctrl)	Key Outcomes/Status
NCT04211766	N/A (38)	CRC Prevention	Exp: Fiber + fish Oil (30 d); Ctrl: Placebo	Serum EPA increased; 12 differentially expressed genes; Feasible
NCT00723398	N/A (266)	Breast Ca. Prev.	Exp: Lovaza (Omega-3) ± Raloxifene	Combo reduced Breast density (28.5 *vs.* 54.3); Lovaza reduced Oxidative stress
NCT00510692	Ph 2/3 (58)	FAP (Polyps)	Exp: EPA (2 g/d); Ctrl: Placebo	EPA reduced rectal polyp count (−12.6%) *vs.* Pbo (+9.7%) (*p* = 0.005)
NCT04548193	Ph 1 (49)	Bladder Ca.	Exp: Cruciferae (Broccoli); Ctrl: F/V Guide	Exp increased Cruciferous intake (1.0 cup/d); Urine ITCs increased
NCT03781778	Ph 2 (10)	CRC	Exp: Resistant starch; Ctrl: Regular starch	RS altered gut flora (decreased *F. prausnitzii*); Feasible
NCT00243022	Ph 2 (12)	High-grade Glioma	Exp: Vit B12 + Boswellia; Ctrl: Vit B12	Trend for reduced brain edema (−2.7 *vs.* +10.2 cc); TTP favored Exp
NCT03140280	Ph 2 (23)	MDS/MPN	Exp: Black Raspberry Powder	Safety: G ≥ 3 SAE in 16.7%; Feasibility study
NCT01465802	Ph 2 (236)	NSCLC (Post-Chemo)	Exp: Probiotic (VSL#3) + Drugs	Prophylactic use reduced G ≥ 2 skin toxicity (23% *vs.* 46%)
NCT03035409	Ph 2 (129)	Metastatic solid Tumors	Exp: Anamorelin + PA + Counseling	Fatigue: Improved FACIT-F & MFSI-SF scores
NCT04205955	Ph 2 (95)	Rectal Ca. Survivors	Exp: Diet Mod Coaching	Bowel: MSK-BFI score slightly higher (30.8 *vs.* 29.5)
NCT00925652	Ph 2 (55)	Breast Ca. (Chemo)	Exp: Diet ± Ex ± Drugs	Lifestyle alone did not sig. improve RFS *vs.* Medical combos
NCT00503776	Ph 2 (41)	Head & Neck Ca.	Exp: Nutr Tx (SNT) ± RT ± Drugs	Similar to above; lifestyle alone showed lower SAEs than drug arms
NCT03314688	N/A (173)	Breast Ca.	Exp: 16x Counseling (Diet + PA); Ctrl: UC	High Chemo completion (94%); Endocrine Tx adherence similar/slightly better
NCT04596384	N/A (398)	Abd. surgery	Exp: Nutr Prehab + Telemonitoring	High CCI rate lower in Exp (7.4% *vs.* 10.4%); Lower attrition
NCT03100409	N/A (137)	Cervical Ca. (RT)	Exp: Low residue diet; Ctrl: SC	Terminated early; Exp had less GI toxicity in interim analysis
NCT02330926	Ph 3 (221)	Cachexia (Adv Ca.)	Exp: n-3 PUFA + Ex + Ibuprofen	Status: Results not reported
NCT00822510	Ph 1 (79)	Prostate Ca.	Exp: Diet Ed + PA + stress Mgmt	Combo arm improved depression, fatigue, & Affect *vs.* Control (*p* < 0.05)
NCT00096330	Ph 1 (20)	CRC prevention	Exp: Folic acid	Status: Results not reported
NCT05387876	N/A (43)	Microbiome (healthy)	Exp: Vitamin D gummies *vs.* Pbo	Status: Active, not recruiting
NCT07272382	N/A (142)	Esophageal Ca.	Exp: Omega-3 drops + PD-1 Inhibitor	Status: In recruiting status
NCT04215367	N/A (21)	CLL (Leukemia)	Exp: High-phenolic EVOO (olive oil)	Status: Results not reported
NCT00555386	N/A (27)	Breast Ca. risk	Exp: Chocolate + soy + selenium	Status: Results not reported
NCT04367493	N/A (45)	Palliative care	Exp: Chocolate (55% or White) for 4 wk	Status: Results not reported
NCT01481584	N/A (28)	Protein Evaluation	Exp: Canola *vs.* Soy Protein Isolate	Status: Results not reported
NCT02324439	Ph 1 (15)	Ovarian Ca. (remiss)	Exp: Flaxseeds (20 g/d) maintenance	Status: Results not reported
NCT00049309	Ph 2 (161)	Prostate Ca. Prev.	Exp: Flaxseed ± Low fat Diet	Status: Results not reported
NCT06880939	N/A (210)	Bladder Ca. (SE)	Exp: Krill oil *vs.* olive oil Placebo	Status: Not yet recruiting
NCT00681512	N/A (6)	NSCLC survivors	Exp: Berry Powder (blue/raspberry)	Status: Terminated (slow accrual)
NCT05852990	Ph 3 (28)	NSCLC (EGFR+)	Exp: Astringent Diet + Glutamine/L.reuteri	Status: In recruiting status
NCT06137248	Ph 2 (76)	CRC (microbiota)	Exp: Astringent Diet + Glutamine/L.reuteri	Status: Results not reported
NCT02696811	N/A (38)	DNA damage	Exp: White Carrots (polyacetylenes)	Status: Results not reported
NCT05907642	N/A (65)	Rectal Ca. (LARS)	Exp: Prunes (100 g/d)	Status: Results not reported
NCT07097155	N/A (120)	General	Exp: Avocado (1/d)	Status: In recruiting status
NCT04066816	N/A (47)	Microbiome	Exp: Ellagic acid (Pomegranate/Walnut)	Status: Results not reported
NCT07144826	Early Ph1	Ovarian Ca. (Chemo)	Exp: Oral Probiotic *vs.* Placebo	Status: Not yet recruiting
NCT06674564	N/A (140)	Laryngeal Ca. (Surg)	Exp: Zinc ± Probiotic *vs.* SOC	Status: Active, not recruiting
NCT00996749	N/A (0)	Prostate Ca.	Exp: Long-term n-3 PUFA	Status: Withdrawn (No accrual)
NCT02069561	N/A (25)	CRC risk	Exp: EPA (2 g/d) for 90d	Status: Results not reported
NCT04857697	Early Ph1	Breast/Lung Ca.	Exp: Preop Probiotic	Status: Results not reported
NCT06892093	N/A (308)	Breast Ca. (Neratinib)	Exp: Probiotics for diarrhea PPX	Status: In recruiting status
NCT07265076	Obs (100)	HCC (Liver Ca.)	Exp: Omega-6 fatty acid caps	Status: Not yet recruiting
NCT03619304	N/A (20)	OSCC (cell line)	Exp: Green/roasted/decaf coffee	Status: *Ex vivo* analysis using patient serum (NR)
NCT01311869	Ph 2 (43)	Uterine fibroids	Exp: EGCG (green tea) 800 mg	Status: Terminated (IRB decision)
NCT06866262	Ph 1/2 (55)	Kidney Ca.	Exp: Inulin gel + Immunotherapy	Status: In recruiting status
NCT01127867	N/A (17)	Obese (menopausal)	Exp: DHA (Omega-3) supplement	Status: Results not reported
NCT01823562	Ph 1 (56)	Prostate Ca. (Preop)	Exp: Black Raspberry (LBR) ± Diet	Status: Active, not recruiting
NCT04310826	N/A (26)	Ovarian Ca. (Chemo)	Exp: Magnesium Intervention	Status: Active, not recruiting
NCT02144649	N/A (45)	Prostate Ca. (Preop)	Exp: Tomato Juice (Tangerine *vs.* Red)	Status: Results not reported
NCT01653925	N/A (120)	Prostate Ca. Prev.	Exp: Dietary fat Mod + dutasteride	Status: Active, not recruiting
NCT07143955	N/A (100)	HCC (Liver Ca.)	Exp: Probiotic + Prebiotic + Diet	Status: Results not reported
NCT04046653	N/A (40)	Prostate Metab.	Exp: Broccoli/Garlic Compounds	Status: Results not reported
NCT05643859	N/A (120)	Benign anorectal	Exp: Broccoli/Garlic Compounds	Status: In recruiting status
NCT00607932	N/A (66)	Prostate Ca. (recur)	Exp: Brassica Veg *vs.* I3C Pill	Status: Results not reported
NCT01950143	N/A (61)	Prostate Ca. Prev.	Exp: Glucoraphanin-enriched Broccoli	Status: Results not reported
NCT01929122	N/A (29)	CRC survivors	Exp: Navy bean powder *vs.* rice bran	Status: Results not reported
NCT01465776	N/A (38)	Oral squamous Ca.	Exp: Black raspberry (LBR) troches	Status: Results not reported
NCT03399331	Ph 1 (45)	Leukemia (mucositis)	Exp: Manuka honey *vs.* EVOO	Status: Results not reported
NCT07242859	N/A (80)	Head & neck (OM)	Exp: Rice bran supplement	Status: In recruiting status
NCT03654638	Ph 2 (25)	Prostate Ca. (ADT)	Exp: Soy bread *vs.* wheat bread	Status: Results not reported
NCT00846313	N/A (0)	CRC	Exp: Indiv diet counseling + ONS/PN	Status: Stopped (lack of resources)
NCT05364359	N/A (40)	Hematological (Tx)	Exp: Taste-Test Tailored counseling	Status: Enrolling
NCT03688646	N/A (40)	Head & neck (RT)	Exp: Intensive Nutr (ONS QD)	Status: Active, not recruiting
NCT04898842	N/A (30)	Bowel obstruction	Exp: 4-stage SBO Diet (RD-led)	Status: Results not reported
NCT05359848	N/A (30)	Chemotherapy Pts	Exp: Adjuvant diet mod	Status: Recruiting
NCT05441163	N/A (50)	Digestive Ca.	Exp: Home Nutr counseling + APA	Status: Recruiting
NCT02481804	N/A (15)	NET (GI symptoms)	Exp: Tailored Diet Mod	Status: Results not reported
NCT04560439	N/A (0)	Breast Ca. Surv.	Exp: METFIT Program	Status: Withdrawn (funding)
NCT02776124	Ph 2 (60)	Nasopharyngeal Ca.	Exp: Diet Counseling + ONS	Status: Results not reported
NCT05959226	N/A (960)	Gastric Ca. (Adv)	Exp: ONS + Exercise	Status: Not yet recruiting
NCT07058571	N/A (30)	Blood Ca.	Exp: Nutr Consult + Exercise	Status: Recruiting
NCT06138223	N/A (196)	Esoph/Gastric Ca.	Exp: Nutr Interv + Protein + Ex	Status: Recruiting
NCT06250686	N/A (185)	Ovarian Ca.	Exp: Nutr Counseling + Exercise	Status: Recruiting
NCT06002269	N/A (8)	Peri-op Lifestyle	Exp: Immunonutrition (Box) + Ex	Status: Results not reported
NCT04329962	N/A (12)	Healthy (Blueberry)	Exp: Blueberry Confections (Metabolism)	Status: Active, not recruiting
NCT05384873	N/A (180)	NSCLC (Immuno)	Exp: High Cal/Pro Immunonutrition	Status: Recruiting
NCT06090916	N/A (100)	Pancreatic Ca.	Exp: Weekly Nutr support + Rx	Status: Active, not recruiting
NCT07057843	N/A (100)	Pancreatic Ca. (CK)	Exp: NRS2002-based sequential Nutr	Status: Results not reported
NCT06595160	Obs (87)	Pancreatic Ca.	Exp: Dietary survey + Fecal specimen	Status: Recruiting
NCT01110980	N/A (120)	Head & Neck (dysphagia)	Exp: Diet Counseling + swallow Tx	Status: Results not reported
NCT07038044	Ph 1 (25)	Solid Tumors	Exp: Arginine-restricted Diet	Status: Not yet recruiting
NCT05115760	N/A (60)	Head & Neck (CRT)	Exp: Pea-Protein ONS	Status: Recruiting
NCT06332664	N/A (158)	Stage IV Ca.	Exp: MDT-led Early Nutr Interv + App	Status: Results not reported
NCT00258401	N/A (11)	Pelvic RT (diarrhea)	Exp: Low residue Diet	Status: Results not reported
NCT03377010	Obs (103)	HSCT survivors	Exp: FFQ + Receptivity survey	Status: Results not reported
NCT02827370	N/A (26)	Breast Ca. (NAC)	Exp: Molecular-Targeted Nutr	Status: Results not reported

Abbreviations: ADT, androgen deprivation therapy; AE/SAE, adverse event/serious adverse event; BMI, body mass index; BWL, behavioral weight loss; CI, confidence interval; CR/CRD, caloric restriction/caloric restricted diet; CRC, colorectal cancer; CT/Chemo, chemotherapy; DASH, Dietary Approaches to Stop Hypertension; DFS/RFS, disease-free survival/recurrence-free survival; DPP, diabetes prevention program; EPA/DHA, eicosapentaenoic acid/docosahexaenoic acid (omega-3 fatty acids); F/V, fruits and vegetables; FMD, fasting-mimicking diet; GI/GL, glycemic index/glycemic load; HbA1c, glycosylated hemoglobin; HEI, Healthy Eating Index; HOMA-IR, Homeostatic Model Assessment of Insulin Resistance; HSCT, hematopoietic stem cell transplant; IF/TRF, intermittent fasting/time-restricted feeding; IGF-1, insulin-like growth factor 1; KD/VLCKD, ketogenic diet/very low carbohydrate ketogenic diet; MedDiet, Mediterranean diet; Met, metformin; MVPA, moderate-to-vigorous physical activity; N, number of participants; N/A, not applicable; NAC, neoadjuvant chemotherapy; NS, not statistically significant; NSCLC, non-small cell lung cancer; Obs, observational; ONS, oral nutritional supplements; OS, overall survival; PA, physical activity; PBO, placebo; PCOS, polycystic ovary syndrome; QoL/HRQoL, quality of life/health-related quality of life; RCT, randomized controlled trial; RD, registered dietitian; RT, radiotherapy or resistance training (context dependent); SOC/UC, standard of care/usual care; WFPBD, whole-foods plant-based diet; WL, weight loss; wk/mo, week(s)/month(s).

### The survivorship interface: Accelerated aging and spatiotemporal symptom resolution

Cytotoxic therapies and obesogenic environments trap cancer survivors in a state of chronic metabolic vulnerability, predisposing them to premature cardiovascular events and secondary neoplasms, particularly in early-onset cohorts. 169, 170, 171, 172, 173 DNA methylation clocks capture this vulnerability, revealing accelerated biological aging and the accumulation of a senescence-associated secretory phenotype (SASP), often paralleled by endocrine decline. 174, 175, 176, 177, 178 Plant-derived bioactive compounds such as epigallocatechin gallate (EGCG) and sulforaphane may partially rescue these aberrant epigenetic landscapes by modulating DNA methyltransferase activity.^179^^,^^180^

In survivorship, the priority shifts from generic dietary counseling to the mitigation of specific, tightly clustered symptoms. 181, 182, 183 Dietary efficacy is highly context dependent: post-diagnosis adoption of anti-inflammatory patterns often reflects compensatory behavior for chronic pain rather than a direct analgesic effect.^184^ For example, the Mediterranean diet (MedDiet) attenuates IL-8-driven fatigue during androgen-deprivation therapy in prostate cancer^185^^,^^186^ but fails to relieve severe fatigue in breast cancer despite improving menopausal symptoms.^187^ Similarly, high-fiber diets or intermittent fasting can improve physical functioning and reduce leukocyte DNA damage, respectively, but may not resolve localized gastrointestinal symptoms or impact objective recurrence endpoints. 188, 189, 190

These findings underscore the need for precision matching, for example, low-fermentable oligosaccharide, disaccharide, monosaccharide, and polyol (FODMAP) diets for osmotic dysregulation versus low-fat regimens for bile acid malabsorption.^191^ In parallel, specialized assessment metrics for cognitive impairment^192^ and biomonitoring are required to prevent substitution-driven micronutrient deficiencies during dietary shifts.^193^^,^^194^ In contrast, uncalibrated supplements such as melatonin fail to resolve fatigue,^195^ and peri-treatment immunonutrition has not prevented radiation-induced mucositis,^196^ highlighting the limits of non-targeted approaches in survivorship care.

### The cachexia conundrum and the multimodal strategy

**Figure 7:**
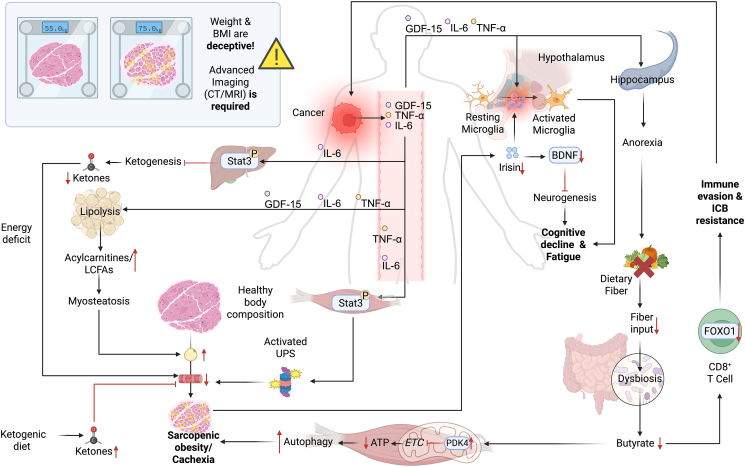
Systemic tumor-driven multi-organ crosstalk in cancer cachexia and the illusion of BMI. Cancer cachexia is a systemic hyper-catabolic syndrome driven by tumor-secreted factors (*e.g.*, IL-6, TNF-α, and GDF-15), which hijack host metabolism across multiple organ systems. (Top Left) Clinical Phenotype: Profound skeletal muscle depletion is frequently obscured by excess adipose tissue and lipid infiltration (myosteatosis), rendering conventional body mass index (BMI) assessments deceptive; thus, advanced imaging (CT/MRI) is required to accurately differentiate healthy body composition from sarcopenic obesity or cachexia. (Metabolic & Muscular Axis): In the liver, IL-6 triggers Stat3 phosphorylation, suppressing hepatic ketogenesis and creating a systemic energy deficit. Ketogenic diets can therapeutically bypass this blockade by supplying alternative ketones directly to the muscle. Concurrently, systemic cytokines and GDF-15 stimulate adipose tissue lipolysis, flooding the circulation with long-chain fatty acids (LCFAs) and acylcarnitines, which deposit in skeletal muscle (myosteatosis). Muscle tissue undergoes severe degradation via a multi-hit mechanism: systemic inflammation activates the ubiquitin‒proteasome system (UPS) via Stat3, while gut dysbiosis-induced butyrate deficiency upregulates mitochondrial PDK4, diminishing ATP production and triggering autophagy. (Gut–Immune Axis): Tumor-derived signals act on the hippocampus to induce severe anorexia. The resulting deprivation of dietary fiber starves the commensal microbiome (dysbiosis), slashing the production of short-chain fatty acids such as butyrate. This butyrate deficit directly downregulates FOXO1 in CD8^+^ T cells, promoting T-cell exhaustion, immune evasion, and resistance to immune checkpoint blockade (ICB). (Muscle–Brain Axis): The brain endures a dual insult: systemic cytokines cross the blood‒brain barrier to activate pro-inflammatory microglia in the hypothalamus, while severe muscle atrophy halts the secretion of the neuroprotective myokine irisin. The resulting plummet in brain-derived neurotrophic factor (BDNF) stifles neurogenesis, culminating in cancer-related cognitive decline and intractable fatigue. Abbreviations: BMI, body mass index; CT, computed tomography; MRI, magnetic resonance imaging; GDF-15, growth differentiation factor 15; IL-6, interleukin-6; TNF-α, tumor necrosis factor alpha; Stat3, signal transducer and activator of transcription 3; LCFAs, long-chain fatty acids; UPS, ubiquitin‒proteasome system; PDK4, pyruvate dehydrogenase kinase 4; ETC, electron transport chain; ATP, adenosine triphosphate; FOXO1, forkhead box O1; ICB, immune checkpoint blockade; BDNF, brain-derived neurotrophic factor.

Balancing therapeutic tumor starvation against host wasting remains a central clinical paradox (Fig. 7). Cancer cachexia (CC) and sarcopenic obesity (SO), where excess adiposity masks profound skeletal muscle depletion,^197^^,^^198^ independently predict chemotoxicity and mortality.^199^^,^^200^ Uncalibrated fasting or ketogenic diets can deplete host NADPH pools and accelerate cachexia in IL-6-producing cancers.^201^^,^^202^ Accordingly, any metabolic restriction should be preceded by objective inflammatory assessment using markers such as the C-reactive protein-to-albumin ratio (CAR) or the Advanced Lung Cancer Inflammation Index (ALI).^203^^,^^204^

In cachectic patients, tumor-derived cytokines (*e.g.*, IL-6, TNF-α) irreversibly activate ubiquitin–proteasome pathways,^205^ ironically mirroring the proteasomal stress that FMDs are designed to induce selectively in tumors.^206^ Effective rescue therefore requires a multimodal strategy that combines pharmacologic blockade of catabolism, microbiome modulation and resistance exercise.^207^^,^^208^ Restoring butyrate-producing *Muribaculum intestinale* mitigates muscle degradation by downregulating pyruvate dehydrogenase kinase 4 (PDK4).^209^ In contrast, leucine-enriched diets paired with exercise promote irisin release, activating the brain-derived neurotrophic factor (BDNF) axis to counter cognitive decline and fatigue. 210, 211, 212, 213, 214, 215, 216 In parallel, antioxidant-rich diets help neutralize microglial activation,^217^^,^^218^ underscoring the need to integrate neurometabolic and musculoskeletal end points into cachexia-focused nutritional oncology.

### Refining nutritional epidemiology by the multi-omic revolution

Nutritional epidemiology is severely bottlenecked by the low spatiotemporal resolution of self-reported food frequency questionnaires (FFQs), which inadequately capture exposures to ultra-processed foods and are subject to severe recall bias.^219^, ^220^, ^221^, ^222^, ^223^, ^224^, ^225^, ^226^, ^227^, ^228^, ^229^ Furthermore, cross-sectional and case–control discrepancies obscure reverse causality and the synergistic effects of complex food matrices.^230^, ^231^, ^232^, ^233^, ^234^, ^235^, ^236^, ^237^, ^238^, ^239^, ^240^ Generic indices frequently mischaracterize cross-cultural diets, demanding a transition toward objective molecular biosensors.^241^, ^242^, ^243^, ^244^, ^245^, ^246^, ^247^, ^248^, ^249^, ^250^, ^251^, ^252^, ^253^, ^254^, ^255^, ^256^, ^257^, ^258^, ^259^, ^260^, ^261^, ^262^, ^263^, ^264^, ^265^, ^266^, ^267^, ^268^, ^269^, ^270^, ^271^, ^272^, ^273^, ^274^

To overcome these limitations, future frameworks must prioritize multi-omic profiling that maps nutritional intake directly onto systemic molecular signatures, enabling the identification of pathogenic lipids (*e.g.*, very long-chain saturated fatty acids [VLCSFAs]) and IgG N-glycosylation patterns.^275^, ^276^, ^277^, ^278^, ^279^, ^280^, ^281^, ^282^ Machine learning explicitly links these signatures to oncological outcomes.^283^, ^284^, ^285^, ^286^, ^287^, ^288^ As comprehensive multi-omics remain cost-prohibitive, AI-driven intermediate biomarkers utilizing standard blood indices, such as CAR, CRP-to-albumin-to-lymphocyte ratio (CALLY), ALI, and neutrophil-to-prealbumin ratio (NPAR), provide practical immunonutritional stratification (Table 3).^203^^,^^204^^,^^289^, ^290^, ^291^, ^292^ However, these algorithms require prospective validation to correct algorithmic biases and flaws, such as the standard body mass index (BMI)'s failure to detect sarcopenic obesity.^198^^,^^289^^,^^293^, ^294^, ^295^ Refined informatics tools are essential for minimizing preprocessing biases and proactively managing host metabolism.^296^, ^297^, ^298^, ^299^, ^300^, ^301^, ^302^

**Table 3:** Summary of transitional immunonutritional biomarkers in oncology.

Biomarker/Index	Calculation formula/Derivation	Predictive efficacy in oncology	Validation stage & current clinical limitations	Reference
CAR (C-reactive protein-to-albumin ratio)	Serum CRP/Serum albumin	Strongly predicts systemic catabolic inflammation, overall survival (OS), and serves as a baseline for cachexia prognosis.	Prospective/Retrospective multi-center. Highly susceptible to non-specific acute phase responses and concurrent transient infections.	203
CCAR (Creatinine-CAR Composite Index)	Integration of Creatinine (Cr) + CAR via random forest machine learning model	Consistently outperforms conventional inflammation/nutrition markers for predicting survival outcomes in cancer cachexia.	Validated in multiple large-scale cohorts (INSCOC database). Requires specific calculation tools/nomograms for real-time clinical use.	203
CALLY (CRP-albumin-Lymphocyte Index)	(Albumin × Lymphocyte count)/CRP	Excellent prognosticator for OS, recurrence-free survival (RFS), and major postoperative complications in patients undergoing curative-intent resections.	Retrospective multi-center. Dynamic fluctuations of lymphocyte counts following cytotoxic treatments complicate continuous monitoring.	292
ALI (advanced Lung Cancer Inflammation Index)	BMI × Albumin/NLR (Neutrophil-to-Lymphocyte ratio)	Accurately predicts mortality and cachexia risk; validated as the optimal inflammatory biomarker for OS in lung cancer and MASLD/MetALD populations.	Retrospective single/multi-center. Critical flaw: BMI component severely confounds the index in patients suffering from hidden sarcopenic obesity.	204,318
NPAR (Neutrophil Percentage-to-albumin ratio)	Neutrophil percentage/Serum albumin	Associated with incident cancer risk (*e.g.*, breast cancer) and predicts all-cause and cardiovascular mortality.	Cross-sectional (NHANES). Readily distorted by clinical corticosteroid administration or acute physiological stress.	291
GLM7 (Glycolipid Metabolism 7 factors)	Machine learning-derived algorithm integrating 7 routine blood tests (glucose, lipid profiles, etc.)	High diagnostic and predictive power for capturing the “Common soil” of multimorbidity, including cancer and cardiovascular diseases.	Retrospective validation (NHANES, CHARLS). Requires external computational platforms; lacks universal threshold standardization across ethnicities.	290
TyG index (Triglyceride-glucose index)	ln[Fasting triglycerides (mg/dL) × fasting glucose (mg/dL)/2]	Predicts increased odds of prevalent breast cancer and higher all-cause/cancer mortality, acting as a robust surrogate for insulin resistance.	Prospective cohort (NHANES). Prognostic value may be modified by age (stronger in <65 yrs) and mediated by oxidative stress/inflammation.	26,319
CTI (CRP-Triglyceride-Glucose Index)	[0.412 × ln (CRP)] + ln [Triglyceride × Glucose/2]	High CTI score is significantly associated with increased 90-day mortality in hospitalized cancer patients.	Retrospective observational study. Requires further prospective, multicenter validation for generalization.	320

Abbreviations: BMI, body mass index; CRP, C-reactive protein; MASLD, metabolic dysfunction-associated steatotic liver disease; NHANES, National Health and Nutrition Examination Survey; OS, overall survival.

### The social exposome and planetary health: Macro-scale determinants of the interface

The social exposome ultimately governs the spatiotemporal dynamics of the host-tumor interface, serving as a cumulative measure of environmental and socioeconomic influences across the life course. Despite established clinical guidelines,^303^ therapeutic adherence is systematically undermined by social determinants of health (SDOH), which dictate the quality and timing of nutritional inputs available to the host. Socioeconomic marginalization and food insecurity function as chronic metabolic stressors, drastically elevating the host's allostatic load. This systemic wear-and-tear triggers a sustained neuroendocrine-immune response, characterized by elevated pro-inflammatory cytokines (IL-6, TNF-α) and chronic cortisol fluctuations. These macro-scale stressors directly offset clinical interventions by creating a pro-tumorigenic macroenvironment. However, structural support, such as medically tailored meals and plant-based micro-substitutions, can bypass individual behavioral barriers, improving compliance and effectively reprogramming the host methylome to reverse SASP-associated senescence.^178^^,^^179^^,^^187^

The host-tumor interface is increasingly compromised by the modern chemical exposome, which operates at the mucosal and endocrine levels. Ultra-processed foods contain emulsifiers and additives that degrade the intestinal mucus layer, facilitating pathobiont translocation and localized inflammation.^17^^,^^304^ Bioaccumulated toxins and per- and polyfluoroalkyl substances (PFAS) act as metabolic disruptors, interfering with nuclear receptor signaling and accelerating carcinogenesis.^305^ Precision nutrition, particularly the maintenance of systemic folate pools (one-carbon metabolism), provides a critical biophysical buffer against this chemical genotoxicity, safeguarding the stability of the host's DNA.^57^

Achieving long-term survival requires a transition to sustainable, systemic frameworks such as the planetary health diet (PHD). The PHD, rich in diverse plant-based fibers and low in processed fats, favorably modulates the host lipidome and restricts the production of pro-tumorigenic secondary bile acids (DCA/lithocholic acid [LCA]). This spatiotemporal shift prevents the recruitment of immunosuppressive Treg cells and limits Wnt/β-catenin hyperactivation.^98^^,^^306^ Ultimately, the deployment of precision nutrition must be matched by structural interventions, such as UPF taxation and culturally aligned whole-food subsidies. These policies are essential to re-engineer the metabolic landscape at a global scale, ensuring that the benefits of precision oncology are not restricted by geographic or socioeconomic boundaries.^307^

## Conclusion

The integration of nutritional science with molecular oncology, immunology, and pharmacology has fundamentally displaced a tumor-centric paradigm in favor of a systems-level architecture. The evidence synthesized here indicates that the host's nutritional state is a primary determinant of tumor evolution, immune surveillance, and therapeutic efficacy. Macroscopic environmental inputs, ranging from dietary patterns to the broader chemical exposome, are funneled through individualized physiological bottlenecks, including the host genome and gut microbiome, and ultimately translated into bioactive metabolites that rewire subcellular machinery, from immune cell epigenetic programming to the biomechanical remodeling of the extracellular matrix.

Dietary-tumor interactions form not a static backdrop but a dynamic, phase-dependent metabolic landscape. The effect of any dietary intervention is constrained by strict spatiotemporal rules. Strategies that transiently weaken tumor defenses during fasting may, if refeeding is not precisely calibrated, provoke an mTORC1-driven oncogenic rebound. As tumors disseminate from the primary site to diverse metastatic niches, their nutritional requirements and fuel preferences shift, necessitating stage- and site-specific metabolic mapping to avoid inadvertently supplying alternative substrates to resistant subclones. Diet should therefore be reconceived not as generic supportive care but as a potent, time-sensitive pharmacological modifier of tumor biology.

Despite conceptual advances, several implementation bottlenecks continue to impede routine clinical translation. Balancing therapeutic tumor starvation against the risk of sarcopenic obesity and cachexia remains a precarious metabolic tightrope. Traditional nutritional epidemiology, dependent on subjective, static questionnaires, lacks the high-resolution, spatiotemporal granularity required to capture real-time host-tumor metabolic flux. At the structural level, socioeconomic inequities and the pervasive chemical exposome frequently render stringent, theoretically optimal regimens infeasible for marginalized populations, creating a compliance gap that widens global health disparities. Overcoming these hurdles will require the fusion of advanced informatics with next-generation molecular biosensors. This emerging paradigm replaces empirical, one-size-fits-all guidance with mathematically optimized, context-specific interventions. Integrating host multi-omic profiles with continuous physiological monitoring, such as wearable continuous glucose monitors (CGMs), will enable *in silico* nutritional trials, allowing clinicians to forecast microbial toxicities and metabolic bottlenecks before they manifest clinically. Composite indices such as CCAR and glycolipid metabolism 7 factors (GLM7) will condense high-dimensional datasets into actionable clinical scores, guiding the precise timing and dosing of diet-mimetic nanotherapies that circumvent the constraints of behavioral adherence.

Ultimately, the implications of precision nutritional oncology constitute a global priority. Embedding targeted metabolic strategies within sustainable frameworks, such as the PHD, offers a path to attenuate the cumulative burden of the chemical exposome and mitigate the accelerated biological aging commonly observed in cancer survivors. Operationalizing this spatiotemporal paradigm in routine practice will require abandoning the low-resolution tools of the past in favor of multi-omic biomarker panels and AI-driven, continuous monitoring. By precisely engineering the host-tumor metabolic interface, modulating the systemic insulin/IGF-1 axis, reprogramming the local microbiome bioreactor, and reshaping subcellular epigenetic circuitry, nutritional oncology can be realized as a rigorously mechanistic pillar of comprehensive cancer survivorship, rather than an adjunctive or empiric afterthought.

## Perspective

The maturation of precision nutritional oncology marks a fundamental paradigm shift from observational dietary advice toward mechanistic metabolic pharmacology. This evolution dictates that the host-tumor metabolic interface be conceptualized as a dynamic spatiotemporal landscape where nutritional inputs act as potent, phase-specific modifiers of tumor evolution and immune surveillance. The future of the field hinges on the integration of chronotherapeutic precision and spatial decoupling. Temporally, future interventions must exploit the tumor's rhythmic vulnerabilities, using transient fasting windows to collapse antioxidant defenses (NADPH/GPX1 axis) while meticulously calibrating the post-fast refeeding phase to prevent mTORC1-driven oncogenic rebound. Spatially, the deployment of bioorthogonal technologies, such as responsive nanomedicines and synthetic biotics, will enable clinicians to physically decouple localized tumor metabolism from host systemic homeostasis, thereby inducing redox catastrophes within the TME without precipitating systemic cachexia. Ultimately, by replacing static dietary guidelines with mathematically optimized models informed by AI-driven digital twins and real-time multi-omic monitoring, nutritional oncology will transcend its role as supportive care. It will emerge as a rigorous mechanistic pillar of comprehensive oncology, capable of rewiring the subcellular epigenetic machinery from m^6^A RNA methylation to histone acetylation and re-engineering the host-tumor interface to fulfill the promise of truly personalized cancer therapy.

## CRediT authorship contribution statement

**Mao Li:** Writing – original draft. **Canhua Huang:** Writing – review & editing. **Shuguang Yu:** Writing – review & editing.

## Funding

This work was supported by grants from the National Key R&D Program of China (No. 2023YFC3402100), the 10.13039/501100001809National Natural Science Foundation of China (No. 82130082, No. 82230127), and the Science and Technology Foundation of Sichuan (China) (No. 2025ZNSFSC0001).

**Box 1 Outstanding questions and future directions in nutritional oncology**

1. Predicting Microbiome–Diet Interactions

How can advanced gut-on-a-chip platforms and dynamic multi-omic profiling be integrated into clinical workflows to forecast an individual patient's risk of adverse microbial shifts before initiating high-fiber or prebiotic interventions?

2. Defining the Refeeding Window

What real-time peripheral biomarkers—such as continuous glucose monitoring combined with circulating exosomal mTORC1 activity or polyamine signatures—are best suited to delineate the physiological limits of the acute post-fast refeeding window and thereby guide the safe use of targeted metabolic inhibitors?

3. Biocontainment of Engineered Biotherapeutics

Can tailored dietary regimens—based on synthetic carbohydrates, restricted lipid configurations, or specific amino acid analogs—be designed and clinically validated as exclusive nutrient sources to control the colonization and activity of engineered live bacterial therapeutics within immunosuppressed TMEs?

4. Standardizing Intermediate Biomarkers

To what extent can international oncology consortia harmonize and deploy machine-learning tools (*e.g.*, models built on dynamic CRP–albumin ratios or composite glycolipid indices) to convert routine blood panels into robust surrogate markers of complex metabolic states, complementing traditional dietary assessments?

5. Validating Planetary Health Diet Frameworks
Can multinational implementation trials quantitatively link plant-forward, planetary health-aligned dietary interventions—especially in marginalized populations—to reductions in epigenetic age (via molecular clocks) and measurable downstream healthcare cost savings?
6. Evolving Regulatory Pathways

How should regulatory agencies (*e.g.*, FDA, EMA) adapt trial design, endpoints, and evidentiary standards to evaluate complex, personalized "food-as-medicine" protocols that lack the patent structure and single-molecule clarity of conventional drugs yet function as potent therapeutic modifiers?

## Conflict of interests

The authors declare that there are no competing interests.
